# Optimizing Engagement With a Smartphone App to Prevent Violence Against Adolescents: Results From a Cluster Randomized Factorial Trial in Tanzania

**DOI:** 10.2196/60102

**Published:** 2025-03-10

**Authors:** Roselinde Janowski, Lucie D Cluver, Yulia Shenderovich, Joyce Wamoyi, Mwita Wambura, David Stern, Lily Clements, G J Melendez-Torres, Lauren Baerecke, Abigail Ornellas, Angelique Nicole Chetty, Jonathan Klapwijk, Laetitia Christine, Ateamate Mukabana, Esmee Te Winkel, Anna Booij, Gervas Mbosoli, Jamie M Lachman

**Affiliations:** 1 Department of Social Policy and Intervention University of Oxford Oxford United Kingdom; 2 Department of Psychiatry and Mental Health University of Cape Town Cape Town South Africa; 3 Centre for Development, Evaluation, Complexity, and Implementation in Public Health Improvement (DECIPHer) School of Social Sciences Cardiff University Cardiff United Kingdom; 4 Wolfson Centre for Young People’s Mental Health Cardiff University Cardiff United Kingdom; 5 Mwanza Research Centre National Institute for Medical Research Mwanza United Republic of Tanzania; 6 Innovations in Development, Education and the Mathematical Sciences (IDEMS) International Reading United Kingdom; 7 Faculty of Health and Life Sciences University of Exeter Exeter United Kingdom; 8 Centre for Social Science Research University of Cape Town Cape Town South Africa; 9 Innovations in Development, Education and the Mathematical Sciences (INNODEMS) Kakamega Kenya; 10 Clowns Without Borders South Africa Cape Town South Africa; 11 Parenting for Lifelong Health Oxford United Kingdom

**Keywords:** digital health, engagement, parenting, adolescents, low- and middle-income country, violence against children, Multiphase Optimization Strategy, randomized factorial experiment, mobile phone

## Abstract

**Background:**

Violence and abuse exert extensive health, social, and economic burdens on adolescents in low- and middle-income countries. Digital parenting interventions are promising for mitigating risks at scale. However, their potential for public health impact hinges on meaningful engagement with the digital platform.

**Objective:**

The objective of this study was to evaluate the impact of 3 intervention design and implementation factors aimed at increasing engagement with a noncommercialized, offline-first smartphone app for caregivers of adolescents in Tanzania, in partnership with the United Nations Children’s Fund, the World Health Organization, and the Tanzanian national government.

**Methods:**

Following Multiphase Optimization Strategy (MOST) principles, we conducted a 2×2×2 cluster randomized factorial trial involving caregivers of adolescents aged 10 to 17 years. Caregivers were recruited by community representatives from 16 urban and periurban communities (ie, clusters) in the Mwanza region of Tanzania. Each cluster was randomized to 1 of 2 levels of each factor: *guidance* (self-guided or guided via facilitator-moderated WhatsApp groups), *app design* (structured or unstructured), and preprogram *digital support* (basic or enhanced). Primary outcomes were automatically tracked measures of engagement (app launches, modules completed, and home practice activities reviewed), with secondary outcomes including modules started, time spent in the app, and positive behaviors logged. Generalized linear mixed-effects models assessed the impact of experimental factors on engagement.

**Results:**

Automatically tracked engagement data from 614 caregivers were analyzed, of which 205 (33.4%) were men. Compared to self-guided participants, receiving guidance alongside the app led to significantly more app launches (mean ratio [MR] 2.93, 95% CI 1.84-4.68; *P*<.001), modules completed (MR 1.29, 95% CI 1.05-1.58; *P*=.02), modules started (MR 1.20, 95% CI 1.02-1.42; *P*=.03), time spent in the app (MR 1.45, 95% CI 1.39-1.51; *P*<.001), and positive behavior logs (MR 2.73, 95% CI 2.07-3.60; *P*<.001). Compared to the structured design, unstructured design use resulted in significantly more modules completed (MR 1.49, 95% CI 1.26-1.76; *P*<.001), home practice activity reviews (MR 7.49, 95% CI 5.19-10.82; *P*<.001), modules started (MR 1.27, 95% CI 1.06-1.52; *P*=.01), time spent in the app (MR 1.84, 95% CI 1.70-1.99; *P*<.001), and positive behavior logs (MR 55.68, 95% CI 16.48-188.14; *P*<.001). While analyses did not detect an effect of enhanced digital support on directly observed engagement, the combination of enhanced digital support and guidance positively influenced engagement across a range of outcomes.

**Conclusions:**

This study is the first to systematically optimize engagement with a digital parenting intervention in a low- and middle-income country. Our findings offer important learnings for developing evidence-based, scalable digital interventions in resource-constrained settings.

**Trial Registration:**

Pan-African Clinical Trial Registry PACTR202210657553944; https://pactr.samrc.ac.za/TrialDisplay.aspx?TrialID=24051

**International Registered Report Identifier (IRRID):**

RR2-10.1186/s12889-023-15989-x

## Introduction

### Background

Violence against children (VAC) has far-reaching individual and societal consequences. Beyond physical harm, violence during childhood negatively impacts brain development and increases risks of poor educational attainment, substance use, and adverse mental and physical health conditions [1,2]. These impacts cascade into broader societal costs, including reduced social participation, unemployment, crime, and intergenerational transmission of violence [2-5], with global economic costs approximating US $7 trillion annually [6].

While VAC occurs in many settings, it is most prevalent within the home environment, where it is typically perpetrated by parents or other caregivers [7]. It spans physical, emotional, and sexual abuse, with disproportionately higher rates in low- and middle-income countries (LMICs), particularly in Africa [8,9]. In Tanzania, national survey data [10] revealed that >70% of respondents aged 13 to 24 years had experienced physical violence from parents, relatives, or guardians before the age of 18 years, including being slapped, kicked, beaten, pushed, or threatened with a weapon. Moreover, approximately 80% of those experiencing sexual violence reported physical violence, while emotional abuse was nearly universal among those who experienced physical violence [10]. A more recent nationally representative study by Nkuba et al [11] found even higher rates, with >90% of secondary school students reporting violent discipline from parents or primary caregivers and 80% of parents acknowledging such practices. This widespread prevalence is associated with limited knowledge of nonviolent parenting alternatives, high caregiver stress, and cultural norms emphasizing child obedience and respect, male honor, and family privacy [10,11].

Parenting interventions, identified by the World Health Organization as a key VAC prevention strategy [12], aim to strengthen parent-child relationships and replace harsh, abusive practices with developmentally appropriate, nonviolent discipline strategies [7]. These interventions are effective in preventing VAC globally [13], particularly when they incorporate skill-building in nonviolent discipline, positive reinforcement, emotion regulation, proactive parenting, and protective relationship promotion [14-16]. However, scaling traditional in-person delivery in LMICs, such as Tanzania, faces significant barriers, including prohibitive delivery costs, extensive human resource requirements, limited geographic accessibility for families, and competing work and childcare demands [17-20].

Digital delivery via smartphone apps offers a promising solution to these implementation and access barriers, particularly in LMIC regions, such as sub-Saharan Africa, where mobile phone penetration is rapidly increasing [21]. The success of mobile health interventions in delivering health care services in sub-Saharan Africa [22] and improving health outcomes across LMICs [23,24] further supports this potential. However, systematic reviews [25-31] show that digital parenting research is almost exclusively concentrated in high-income countries (HICs), with interventions predominantly focusing on child behavior management rather than violence prevention. Moreover, these interventions typically experience poor participant engagement [32], defined as both the extent of program usage (eg, amount, frequency, duration, and depth) and subjective user experience (eg, attention, interest, and affect) [33].

Poor engagement undermines an intervention’s impact at both individual and population levels. At the individual level, participants fail to acquire essential skills needed to prevent VAC and improve child well-being, while at the population level, governments and policy makers are unlikely to implement programs widely when only a small proportion of participants engage with and benefit from them [34]. Consequently, poor engagement severely limits the potential public health impact of digital parenting interventions [35,36]. Understanding and optimizing engagement in LMIC contexts is thus critical for establishing digital parenting interventions as a scalable violence prevention strategy.

Existing studies examining engagement with digital parenting interventions in HICs have largely been descriptive [37,38] or focus on sociodemographic and behavioral characteristics associated with engagement [39-42]. A recent review of technology-assisted parenting interventions has advanced this literature by identifying several potential engagement-promoting components, including practical technology support, interactive program features, tailoring, and control features [43]. Similar components have been identified across the broader mobile health literature, such as personalized content and feedback [44-46], user-friendliness [47], a visually appealing layout and the integration of a tutorial on how to use the app [44], human guidance [48-50], peer-based asynchronous communication features [51], and reminders [33,44,52]. However, experimental evidence for these components remains limited; to date, only 1 known trial has experimentally evaluated engagement strategies for digital parenting interventions [53], with no such trials conducted in LMICs. This gap is particularly significant given that digital interventions and their delivery strategies comprise multiple components, each with potentially different effects on engagement. Furthermore, findings from HICs may not generalize to LMIC settings, such as Tanzania, where technological infrastructure, digital literacy, and cultural contexts differ substantially.

### Objectives

In response to the need for scalable violence prevention in LMICs, the Global Parenting Initiative, a 5-year research-within-implementation collaboration between universities, foundations, and implementing partners, is developing and evaluating open-source digital parenting programs based on the Parenting for Lifelong Health (PLH) intervention suite. One of these interventions is ParentApp, a smartphone app designed to address adolescent exposure to VAC. Using the Multiphase Optimization Strategy (MOST) framework [54] for systematic intervention optimization, we conducted a cluster randomized factorial trial to identify optimal components for maximizing engagement with ParentApp among socioeconomically disadvantaged caregivers of adolescents in Tanzania. The trial examined 3 factors: *guidance, app design*, and *digital support*. Our primary objective was to determine the independent effects of these factors on primary and secondary engagement outcomes. An exploratory objective was to identify potential interaction effects between the factors.

## Methods

### Trial Design

The trial used a cluster randomized factorial design with three experimental factors: (1) *guidance* (self-guided or guided; factor A), (2) *app design* (structured or unstructured; factor B), and (3) *digital support* (basic or enhanced; factor C). Each factor was varied at 2 levels (lower dose or inactive vs higher dose or active), yielding an 8-condition (2×2×2) trial. A full factorial design was used, allowing all clusters to potentially be randomized to all combinations of factors and their corresponding levels. Further details on the study design are provided in the published protocol [55]. The trial was preregistered on the Pan-African Clinical Trial Registry (PACTR202210657553944) on October 11, 2022. The CONSORT (Consolidated Standards of Reporting Trials) 2010 extension for randomized factorial trials [56] and the CONSORT-EHEALTH (Consolidated Standards of Reporting Trials of Electronic and Mobile Health Applications and Online Telehealth) checklist [57] were used for reporting.

### Setting and Participants

Target communities were urban and periurban sub-wards characterized by high rates of poverty in the Mwanza region, Tanzania. Eligible clusters were sub-wards that (1) were located within 3 selected low-income wards, (2) had no previous involvement in ParentApp testing, and (3) contained at least 40 smartphone-owning households. Within each cluster, participants were primary caregivers of at least 1 adolescent aged 10 to 17 years. Caregivers were eligible if they (1) were aged ≥18 years, (2) lived in the same household as their adolescent for at least 4 nights per week in the previous month, (3) had regular access to an Android smartphone, and (4) provided written informed consent. Participants were excluded from the study if they were unable to read or had a severe learning disability that affected their ability to provide informed consent.

### Recruitment, Enrollment, and App Onboarding

Following a 2-week community mapping period conducted by Tanzania’s National Institute for Medical Research (NIMR) in October 2022, the fieldwork team, in collaboration with Tanzania-based nongovernmental organization (NGO) Investing in Children and Strengthening Their Societies (ICS), initiated recruitment procedures in 3 low-income wards. Centralized stakeholder meetings were held in 2 wards, while in the third ward, sub-ward-level consultations took place due to logistical constraints. A total of 31 community leaders, including ward executive officers, community development officers, and local representatives, were briefed about the study. Those who agreed to support the study were tasked with identifying and contacting potential eligible families within their respective sub-wards over an 8-week recruitment period. Community leaders provided standardized information about the study purpose and intervention content and invited interested families to attend orientation and onboarding sessions.

These sessions were organized by study cluster and held at accessible locations, such as schools and community centers. Sessions were cofacilitated by NIMR research assistants and ICS facilitators. Families first attended a group orientation where they received detailed information about the program objectives, study procedures, data collection requirements, and time commitments. Research assistants then conducted one-on-one eligibility screening using an Open Data Kit checklist. Written informed consent was obtained from caregivers who met all eligibility criteria.

The subsequent app onboarding lasted approximately 90 minutes and was conducted in small groups of 5 to 10 participants. This included guided assistance with app installation via Google Play (Google LLC), technical troubleshooting, and an orientation to app features. Facilitators demonstrated key functionalities, such as module navigation, activity completion, and resource access. Participants then practiced these skills by completing the app’s first module, which concluded with an integrated baseline assessment.

### Intervention

#### Overview

ParentApp is a smartphone app adapted from PLH Teens, an in-person, group-based program originally developed in South Africa to address the need for cost-effective, culturally appropriate violence prevention [58]. Initial evaluations of PLH Teens demonstrated significant improvements in positive parenting practices, along with reductions in child maltreatment, parental depression, stress, substance use, and financial stress [58]. PLH Teens has since been adapted and widely implemented across >18 LMICs [59], including a large-scale delivery in Tanzania to >75,000 caregivers and adolescents [60]. Building on this strong, emerging evidence, ParentApp was developed to address access and scalability challenges associated with in-person delivery. The development and delivery of ParentApp in Tanzania were jointly led by the Universities of Oxford and Cape Town; PLH; Innovations in Development, Education and the Mathematical Sciences; Clowns Without Borders South Africa; NIMR; and ICS, in collaboration with the United Nations Children’s Fund, the World Health Organization, and the Tanzanian national government. The intervention content is described in accordance with the TIDieR (Template for Intervention Description and Replication) checklist (Multimedia Appendices 1 and 2).

#### ParentApp Development

ParentApp underwent systematic development through four phases: (1) initial adaptation and codevelopment with researchers, program specialists, technical experts, and PLH Teens participants in South Africa; (2) user testing with 24 participants across 9 African countries [61]; (3) feasibility testing of self-guided delivery with 107 caregivers in South Africa; and (4) pre- and postpilot testing with 103 caregiver-adolescent dyads in Tanzania, which included remote guidance via phone calls or WhatsApp (Meta Platforms, Inc). Iterative refinements to content, design, and delivery approach across these phases produced the version of ParentApp evaluated in this trial.

#### ParentApp Design and Content

ParentApp is an open-source, offline-first Android app (compatible with version 5.5.1 or later) comprising 12 modules that integrate text, images, and audio to deliver a condensed version of the in-person program (Table 1). The introductory module focuses on parental self-care and stress reduction, concluding with a welcome survey that collects baseline data and enables users to select their preferred delivery format (individual or group) and engagement schedule. The core intervention consists of 10 modules covering evidence-based practices shown to effectively reduce harsh and abusive parenting [7]. A final module consolidates learning and includes a postintervention assessment.

Each module uses recurring activities to reinforce learning and practical skill building. Modules begin with mindfulness activities featuring emotional check-ins and relaxation exercises, followed by comics that illustrate positive and negative caregiver-adolescent scenarios with reflection questions. Audio testimonials from Tanzanian parents share benefits and experiences, while *Essential Tools* summaries provide quick access to key skills and examples. Modules conclude with home practice assignments that include structured activities and a follow-up *Home Practice Review* for activity reflection. Throughout the app, users can log their skill implementation through *ParentPoints*, a behavioral tracking system for monitoring 10 evidence-based practices that promote positive parenting and mental health (Multimedia Appendix 2). Beyond module activities, users have access to a library with essential parenting tips, local support resources, and technical support. To encourage ongoing engagement, automated push notifications are triggered at 1, 6, and 30 days following the user’s last app launch.

**Table 1:** ParentApp module content and learning objectives.

Module	Core skills and objectives	Home practice^a^
1. Parental self-care and stress reduction	Developing essential mental and physical well-being skills to strengthen parents’ capacity to support their families	Practice daily relaxation techniques, identify moments of achievement, and reward successes.
2. One-on-one time	Spending meaningful time with adolescents, using active listening to foster trust, communication, and positive relationships	Schedule and spend 5 to 20 min of focused time with adolescents daily.
3. Praise and positive reinforcement	Using effective praise techniques to encourage positive behavior in adolescents	Practice praising adolescents daily.
4. Positive instructions	Providing clear, specific, and positive directions to improve communication and cooperation	Use positive instructions with adolescents daily.
5. Managing stress	Enhancing emotional awareness and communication skills to support mental well-being and reduce the risk of violent reactions during challenging situations	Practice breathing exercises before reacting.
6. Family budgeting	Identifying strategies for managing and saving money to reduce financial stress and establish achievable family goals	Create a budget with adolescents and other family members.
7. Establishing rules and routines	Engaging adolescents in establishing household rules and routines to foster compliance and build a safe, supportive, and consistent home environment	Practice making rules together with adolescents.
8. Consequences and accepting responsibility	Promoting shared parent-adolescent responsibilities; applying realistic, appropriate consequences for noncompliance; and emphasizing positive discipline over punitive measures	Discuss negative and positive consequences with adolescents.
9. Problem-solving	Building family collaboration skills to identify, address, and resolve challenges through joint problem-solving	Teach adolescents 4-step problem-solving: know it, solve it, try it, and test it.
10. Teen safety in the community and online	Mapping risks in community and online environments to establish protective rules for adolescents	Map safe and unsafe spaces with adolescents and create a family safety plan.
11. Dealing with crisis	Remaining calm in crisis situations and developing immediate and long-term plans to respond effectively, using available support services	Discuss crisis scenarios with adolescents and create family crisis response plans.
12. Celebration and next steps	Reflecting on key learning and solidifying plans for ongoing peer support	Review key skills learned.

^a^Modules 2 to 11 include home practice assignments with follow-up reviews for activity reflection.

### Experimental Factors

#### Overview

Table 2 presents an overview of the core features associated with each factor level. Justification for the selection of factors is also provided in the published protocol [55].

**Table 2:** Experimental factors, levels, and their core features.

Factor		Description	Core features
**Guidance**			
	Self-guided	Participants accessed ParentApp’s full content throughout the study.	ParentApp without human support
	Guided	Participants accessed ParentApp’s full content throughout the study and received WhatsApp group guidance.	ParentApp WhatsApp groups moderated by 1 lead facilitator and 1 cofacilitator Facilitators monitored discussions and shared tips and reminders Facilitators led a weekly 1-hour live-chat session per group
**App design**			
	Structured	ParentApp content followed a sequential, weekly format.	12 modules First module available immediately, with 1 new module unlocked every 7 days Sequential module order Content-related notifications Images featuring generic blue amorphous cartoon characters
	Unstructured	ParentApp content followed an open, nonsequential format.	12 modules All modules available immediately Nonsequential module order Modules divided into shorter tasks and activities Content-related notifications disabled Culturally adapted images featuring humanlike figures
**Digital support**			
	Basic	Participants received an in-app navigation tour at first app launch, followed by a group-based orientation of the app.	Brief built-in app navigation tour showing core app features Group orientation on core features led by trained research assistants
	Enhanced	Participants received an in-app navigation tour at first app launch, followed by a group-based orientation of the app and a supplementary training session focusing on broader smartphone skills.	Brief built-in app navigation tour showing core app features Group orientation on core features led by trained research assistants Supplementary group training (15 to 20 min) on general smartphone skills led by trained research assistants

#### Factor A: Guidance (Self-Guided or Guided)

During formative testing, WhatsApp groups showed promise for enhancing app-based delivery. In this trial, half of the clusters received no human support after app onboarding, while the remaining half received guidance via facilitator-moderated WhatsApp groups. The purpose of these groups was to promote social connection and sustain engagement with ParentApp. Each cluster in the guided condition had a corresponding WhatsApp group, overseen by a female-male facilitator pair (totaling 8 groups and 4 facilitator pairs). Following a standardized manual, facilitators shared tips and reminders, monitored discussions, addressed questions, and led weekly 1-hour live-chat sessions to encourage discussion and reflection. The facilitators, who were locally recruited paraprofessionals with previous experience supporting caregivers remotely during the Tanzanian pilot, received training from Clowns Without Borders South Africa, an NGO experienced in training PLH program facilitators.

#### Factor B: App Design (Structured or Unstructured)

**Figure 1:**
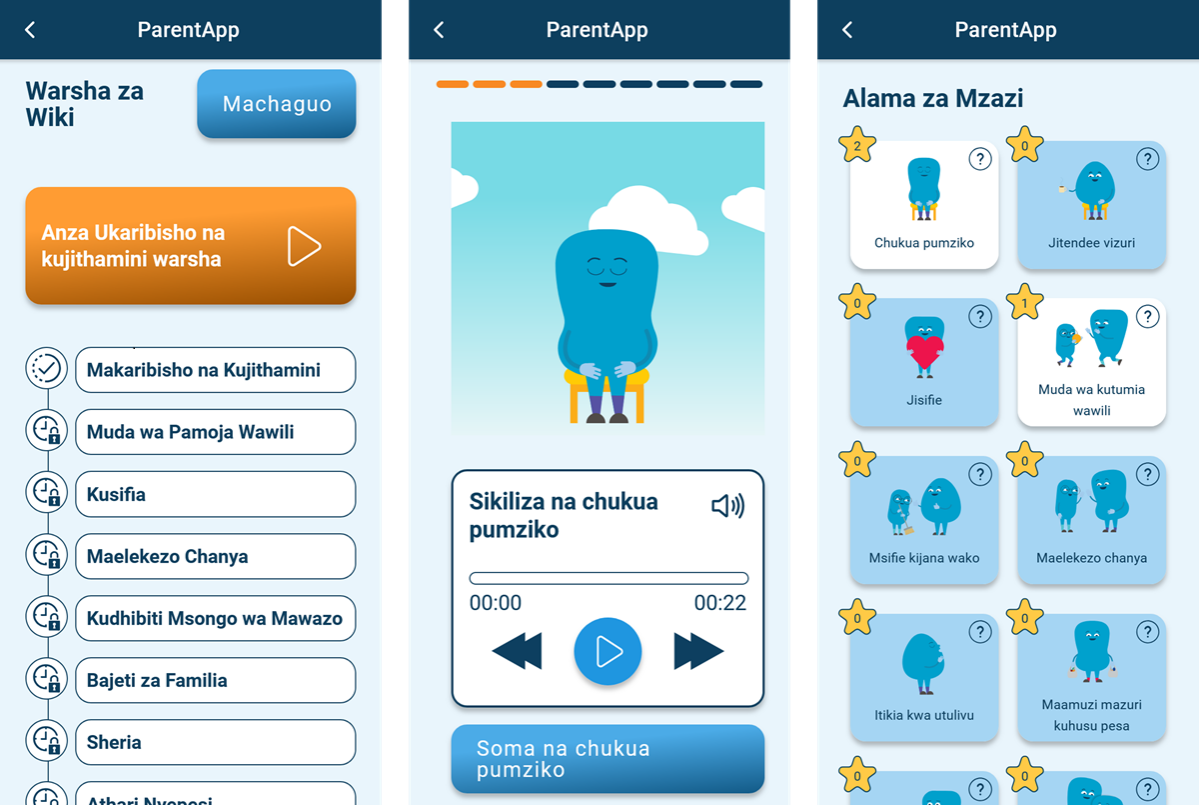
Screenshot examples of the structured app design with generic illustrations.

**Figure 2:**
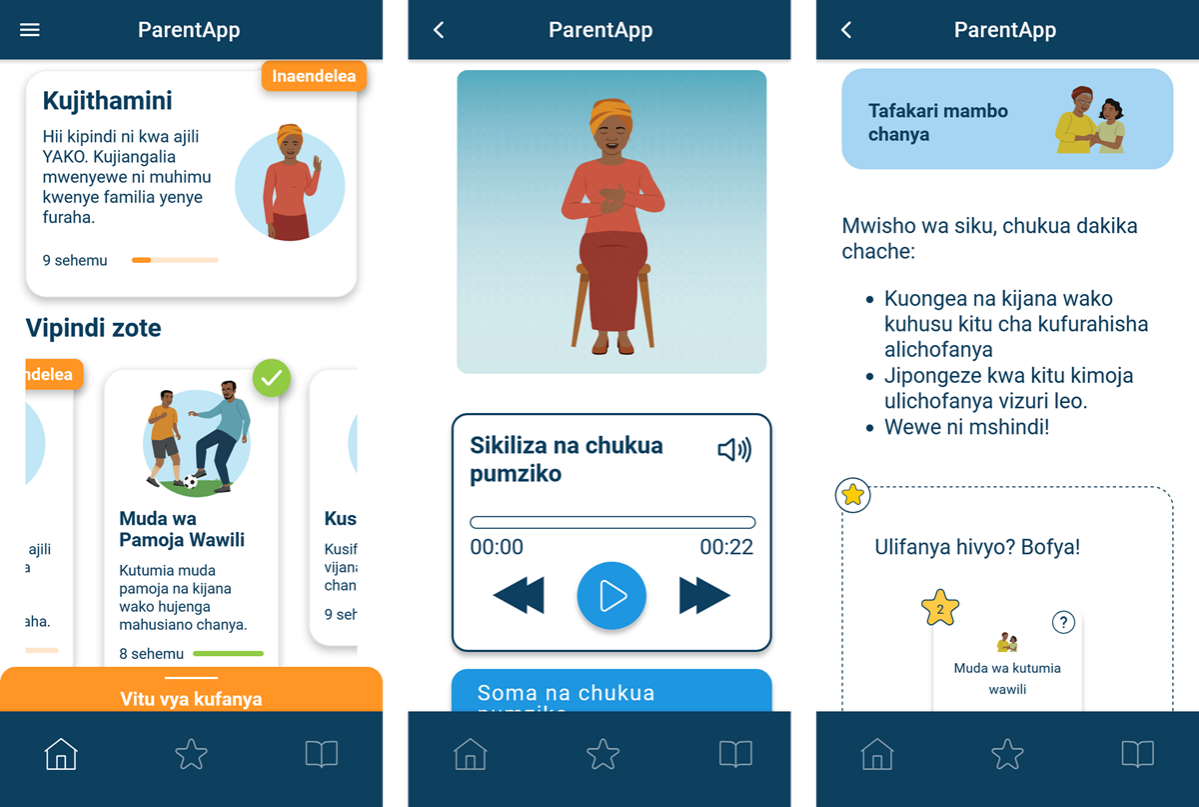
Screenshot examples of the unstructured app design with culturally adapted illustrations.

ParentApp was originally designed with a sequential progression to match PLH Teens’ learning objectives and time frame. The original design also featured generic blue cartoon characters intentionally designed to facilitate global implementation. However, early testing highlighted 2 potential areas for improvement: some participants found the fixed weekly module releases limiting, while others questioned the acceptability of the generic illustrations. In response, this trial evaluated 2 versions of the app’s design (refer to Figures 1 and 2 for screenshot examples). Half of the clusters received the structured design, which maintained sequential module delivery at 7-day intervals over 12 weeks. This version included automated content notifications aligned with module releases, scheduled unlocking of home practice reviews for modules 2 to 11 at the end of each 7-day cycle, and the original generic illustrations. The remaining clusters received the unstructured design, which offered immediate access to all modules and activities after installation. This version incorporated several modifications: users could navigate the 12 modules at their own pace and in their preferred sequence; modules were subdivided into shorter tasks for easier navigation, with home practice review as the final task for modules 2 to 11; automated content notifications were disabled to accommodate flexible access; and generic cartoon images were replaced with culturally adapted illustrations featuring humanlike characters tailored to the Tanzanian context.

#### Factor C: Digital Support (Basic or Enhanced)

During formative testing, digital literacy challenges emerged as a critical consideration for app engagement. Consequently, this trial evaluated 2 levels of digital support during the onboarding session. Basic support, offered to all clusters, included an embedded ParentApp navigation tour followed by a group-based orientation of the app with demonstrations and practice opportunities. Half of the clusters received enhanced support, which included an additional 15- to 20-minute session focused on improving general smartphone literacy and user confidence. This supplementary training was developed by Innovations in Development, Education and the Mathematical Sciences Kenya, an NGO providing technical support during trial implementation. The training covered essential smartphone skills, including app installation, effective internet use, and online safety, and was delivered by research assistants at the end of the onboarding session. Both levels of digital support were optional.

### Measures and Assessment

#### Overview

Engagement was operationalized across three phases: (1) initial, (2) ongoing, and (3) quality, aligning with established digital intervention literature [33,43,62,63]. Engagement data were collected through automated tracking each time a participant interacted with ParentApp. Use records, including module completion rates, content viewing patterns, and responses to in-app tasks and the baseline assessment, were uploaded to a Metabase cloud server (Metabase, Inc) whenever a device accessed the internet. Additional use data were automatically captured through Matomo Analytics (InnoCraft Ltd), including visit timestamps, session duration, and the frequency of actions or pages viewed. Data monitoring protocols were implemented to ensure accurate tracking and verify that participants received the correct app version based on their cluster allocation. The final dataset was extracted from Metabase and Matomo Analytics in March 2023, approximately 4 months after the last cluster of participants enrolled in the study. Research assistants and facilitators reported estimated attendance rates and session durations for the enhanced digital support training and WhatsApp live-chat sessions, respectively.

#### Primary Outcomes

Three primary engagement outcomes were evaluated: (1) the number of *app launches* throughout the intervention period (initial engagement), (2) the number of *modules completed* out of 12 (ongoing engagement), and (3) the number of *home practice activities reviewed* out of 10 (quality of engagement). The home practice review outcome underwent a post hoc modification from the protocol, evaluating user initiation of postactivity reviews rather than access to the home practice tab for activity instructions.

#### Secondary Outcomes

Secondary engagement outcomes included (1) the number of *modules started* out of 12 (initial engagement), (2) the total *time spent in the app* during the intervention period (ongoing engagement), and (3) the number of self-reported *positive parenting and mental health–promoting behaviors logged* throughout the intervention period (quality of engagement).

#### Baseline Sociodemographic and Behavioral Measures

Baseline demographic and behavioral data were collected using a self-administered questionnaire embedded within the app’s welcome survey in the first module. The questionnaire included multiple response formats: demographic questions primarily used binary choice buttons (eg, gender: woman or man) or number entry fields (eg, age), while behavioral measures used frequency scales. Participants could navigate between pages to review and modify their responses, with the assessments taking approximately 10 to 15 minutes to complete. All behavioral measures were nonproprietary, freely available, and recommended by the Global Parenting Initiative. Given the trial’s focus on engagement rather than caregiver outcomes, abbreviated scales were used to minimize participant burden. All measures were translated into Kiswahili and back translated and were previously used in Tanzania [60].

Demographic variables included caregiver age and gender, household structure, orphanhood status, and 2 items adapted from the Financial Self-Efficacy Scale on financial stress and food insecurity (1 item each) [64]. Child maltreatment was assessed using 4 items from the International Society for the Prevention of Child Abuse and Neglect Screening Tool-Trial Version [65]. This included subscales for physical abuse (2 items; eg, “How many times in the past month did you hit, spank or slap your child with a hand or object?”) and emotional abuse (2 items; eg, “How many times in the past month did you shout, scream or yell at your child?”), rated on a 0 to ≥8 frequency scale. Positive parenting was measured using 5 items from the Alabama Parenting Questionnaire [66], assessing involvement (2 items; eg, “How many times in the past month did you praise your teen?”) and supervision (3 items; eg, “How many times in the past month did your teen stay out past their curfew?”), rated on a 0 to ≥8 frequency scale. Parental depression was measured using 3 items from the Centre for Epidemiologic Studies Depression Scale [67]. Items were rated on a 0- to 7-day frequency scale (eg, “How often did you feel that everything you did was an effort?”). Parenting stress was assessed using 1 item adapted from the Parental Stress Scale [68], “How many times in the past month did caring for your children make you feel very stressed?” rated on a 0 to ≥8 frequency scale.

### Power

An a priori simulation–based power analysis determined that 640 caregivers across 16 clusters (40 per cluster) were needed for adequate statistical power [55]. This cluster size was selected for future scalability, as larger WhatsApp groups offer greater reach and cost-effectiveness. Simulations accounted for clustering at the sub-ward level, participant-level covariates, and an α level of .05.

### Randomization

The factorial experiment was conducted at the cluster level for logistical efficiency, streamlining screening, enrollment, and baseline data collection for participants receiving the same experimental conditions. Community representatives compiled lists of potentially eligible families within their designated sub-wards. Fieldwork coordinators then formed clusters by grouping geographically proximate sub-wards to minimize the risk of cross contamination. An off-site research team member performed single-stage randomization, assigning each eligible cluster to 1 of the 8 experimental condition combinations using a randomization algorithm in R (R Foundation for Statistical Computing). Final cluster sizes ranged from 32 to 49 caregivers (mean 38.38, SD 6.20).

### Blinding

Blinding was not feasible for the in-country research team and facilitators due to their involvement in implementation. Participants were only informed about the experimental conditions to which their cluster was assigned. The data analyst was blinded to cluster allocation but not to condition assignment due to effect coding [69].

### Statistical Analysis

Analyses used generalized linear mixed-effects models with a Poisson distribution to estimate the impact of experimental factors on engagement. Models included a random intercept term to account for the nesting of participants within clusters. Intention-to-treat analyses were conducted but included only participants who successfully installed ParentApp, as data collection was contingent upon app installation. Supplementary analyses found no significant effect of cluster randomization to experimental conditions on app installation status (Table S1 in Multimedia Appendix 3). Missingness in engagement outcomes was minimal, affecting only 2 variables: app launches (6/614, 1%) and time spent on the app (11/614, 1.8%). Multiple imputation with fully conditional specification [70] was used to address missingness in caregiver baseline data (5% to 7.5% at item level). A total of 10 imputations were conducted using predictive mean matching. Clustering was accounted for by specifying –2 in the predictor matrix. Imputation at the item level was passive, summing behavioral items to generate total scores within the imputation process. Following imputation, caregiver baseline data were sample-mean centered within each of the 10 imputed datasets. Analyses were conducted in R (version 4.3.1), using the glmer function from the *lme4* package for generalized linear mixed-effects models [71], and imputations were performed using the *mice* package [72].

In primary analyses, each factor’s individual effect on primary and secondary outcomes was evaluated separately. Models were adjusted for the prespecified covariates: caregiver age, caregiver gender, financial stress, food insecurity, child maltreatment (physical and emotional abuse), positive parenting involvement and supervision, parental depression, and parenting stress. In exploratory analyses, all 3 factors were included as main effects alongside their 2-way interactions, while adjusting for baseline characteristics. The inclusion of interaction terms followed recent recommendations for factorial designs to account for potential interdependencies between factors [73]. All main and 2-way interaction effects were estimated using the full sample. Assuming similar effect sizes, this provided equally high power to detect all effects [54]. Estimates for each model were pooled across the 10 imputed datasets using Rubin’s rules [72]. Factors were effect-coded, assigning –1 to inactive or lower-dose levels and 1 to active or higher-dose levels (Multimedia Appendix 4). This coding scheme calculates the difference between levels as (+1 – (–1) = 2), whereas with dummy codes, it is simply (1 – 0 = 1) [69]. To ensure an accurate representation of the effect of a 1-unit change in the predictor variable within the effect coding scheme, unstandardized regression coefficients and their SEs were multiplied by a scaling constant of 2. Poisson regression coefficients (which represent the log of the expected change in the outcome per 1-unit increase in the predictor while holding other variables constant) and 95% CIs were exponentiated for easier interpretation. The main effects were interpreted as the ratio of mean event counts or mean ratio (MR), comparing participants in clusters randomized to the higher-dose factor level to those randomized to the contrasting factor level. Interaction terms were interpreted as the additional effect of 1 factor level in the presence of another.

### Ethical Considerations

Ethics approval was granted by NIMR (NIMR/HQ/R.8s/Vol.IX/3856) and the University of Oxford Departmental Research Ethics Committee (R69744/RE001). Written informed consent was obtained from all study participants at the onboarding session. Participants also accepted the app’s terms and conditions and privacy policy, which detailed how personal information from ParentApp would be collected, used, and shared. After completing the onboarding session, participants received a US $2 honorarium and monthly 1 GB internet bundles for 4 months to support data synchronization with study servers and WhatsApp group participation where applicable. No additional incentives or compensation were provided.

## Results

### Participant Flow

**Figure 3:**
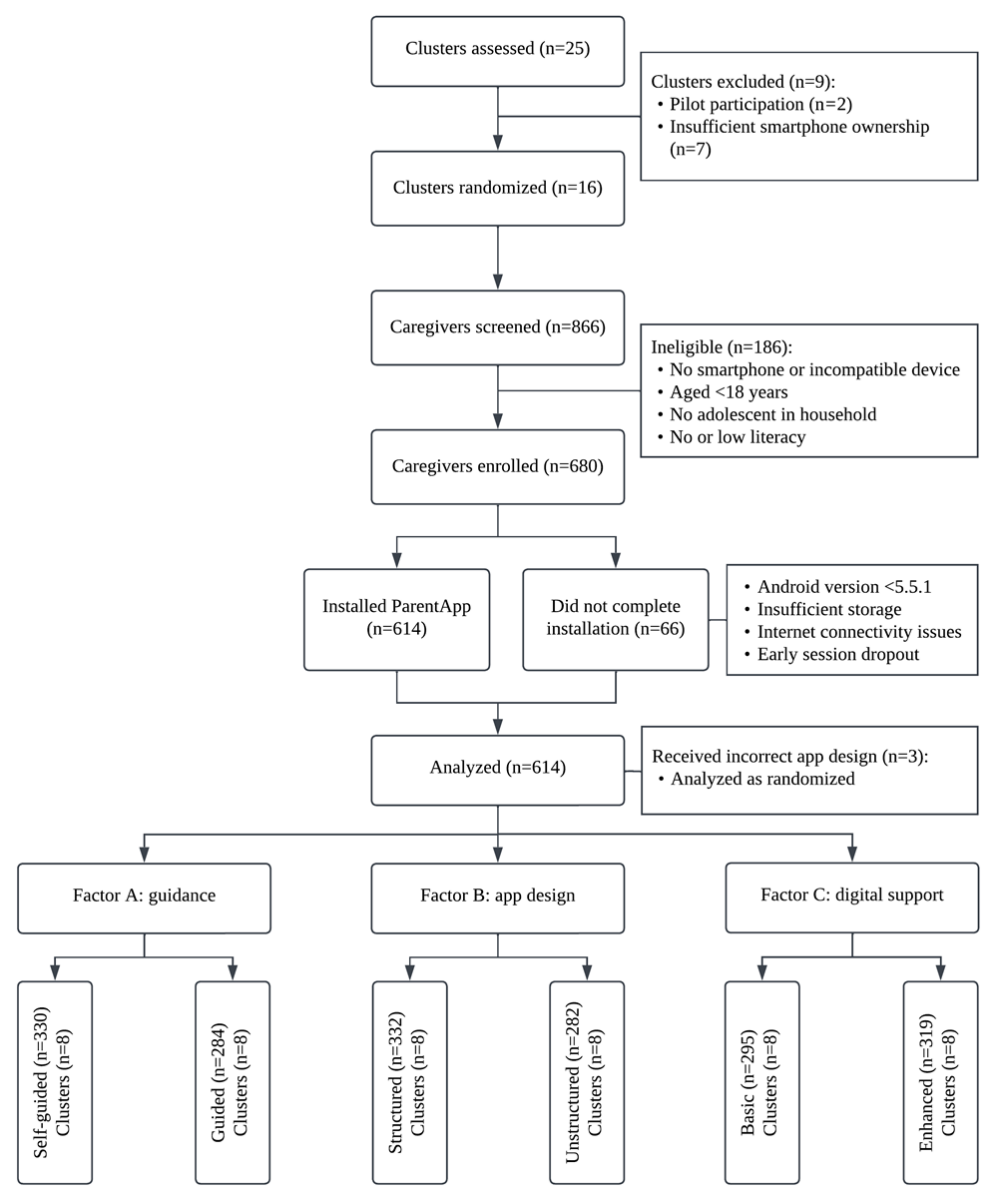
CONSORT (Consolidated Standards of Reporting Trials) diagram of participant flow through the 2×2×2 factorial trial.

Participant recruitment and onboarding activities began on October 24, 2022, and concluded on December 1, 2022, coinciding with Tanzania’s short rainy season. Of 866 caregivers screened for eligibility, 680 (78.5%) met the inclusion criteria and consented to participate. Among enrolled participants, 66 (9.7%) did not complete app installation due to technical difficulties, including phone-to-app incompatibility, insufficient storage capacity, and internet instability, while others left the onboarding session prematurely due to poor weather conditions and scheduling conflicts. The final sample included 614 participants (90.3% of those enrolled) who successfully installed the app, all of whom were included in intention-to-treat analyses. A CONSORT diagram illustrating participant flow through the study is presented in Figure 3.

### Participant Characteristics

Participant baseline characteristics, summarized in Table 3, showed a comparable distribution across the 3 factors’ levels and the overall sample. Caregiver age ranged from 18 to 75 (mean 35.94, SD 11.84) years. One-third (205/614, 33.4%) of the caregivers were men, which is notable given the challenges parenting programs typically face in engaging fathers [74]. Households comprised an average of 3 (SD 1.80) adults and 3.61 (SD 2.12) children aged between 0 and 17 years. Most caregivers (485/576, 84.2%) reported experiencing food insecurity at least once in the past month, with 35.1% (202/576) reporting ≥7 instances during this period. Nearly half (276/576, 47.9%) of the sample reported that their adolescents had experienced the loss of a primary caregiver during their lifetime. Among these, 72.1% (199/276) reported that the death had occurred within the past 3 years, a trend likely influenced by the COVID-19 pandemic.

**Table 3:** Baseline characteristics for the full sample and each factor level (N=614).

Characteristic			Total^a^		Factor A: guidance				Factor B: app design				Factor C: digital support		
					Self-guided		Guided		Structured		Unstructured		Basic		Enhanced
**Caregiver, n**			n=577		n=307		n=270		n=307		n=268		n=281		n=296
	Age (y), mean (SD)	35.94 (11.84)		35.83 (12.09)		36.07 (11.56)		37.16 (11.54)		34.54 (12.04)		36.35 (11.67)		35.55 (12.00)	
**Gender^b^**			n=614		n=330		n=284		n=332		n=282		n=295		n=319
	Women, n (%)	409 (66.6)		217 (65.8)		192 (67.6)		244 (73.5)		165 (58.5)		193 (65.4)		216 (67.7)	
	Men, n (%)	205 (33.4)		113 (34.2)		92 (32.4)		88 (26.5)		117 (41.5)		102 (34.6)		103 (32.3)	
**Household size^c^**			n=580		n=310		n=270		n=313		n=267		n=281		n=299
	Overall, mean (SD)	6.60 (3.23)		6.55 (3.53)		6.66 (2.85)		6.70 (3.46)		6.48 (2.95)		6.46 (3.16)		6.73 (3.30)	
	Adults, mean (SD)	2.99 (1.80)		3.04 (1.99)		2.94 (1.57)		3.06 (2.04)		2.91 (1.49)		2.9 (1.71)		3.05 (1.89)	
	Children (aged 0-17 y), mean (SD)	3.61 (2.12)		3.51 (2.29)		3.72 (1.90)		3.64 (2.16)		3.57 (2.07)		3.53 (2.16)		3.68 (2.08)	
**Food insecurity**			n=576		n=310		n=266		n=308		n=268		n=277		n=299
	≥1 day in the past month, n (%)	485 (84.2)		267 (86.1)		218 (82)		258 (83.8)		227 (84.7)		235 (84.8)		250 (83.6)	
	≥7 days in the past month, n (%)	202 (35.1)		113 (36.5)		89 (33.5)		108 (35.1)		94 (35.1)		103 (37.2)		99 (33.1)	
**Orphanhood^d^**			n=576		n=308		n=268		n=306		n=270		n=277		n=299
	Lifetime, n (%)	276 (47.9)		160 (51.9)		116 (43.3)		153 (50)		123 (45.6)		137 (49.5)		139 (46.5)	
	Past 3 years, n (%)	199 (72.1)		117 (73.1)		82 (70.7)		108 (70.6)		91 (74)		95 (69.3)		104 (74.8)	

^a^Sample sizes vary due to item nonresponse.

^b^Missing caregiver gender data were supplemented with information from the screening questionnaires, resulting in 614 complete observations.

^c^Cases missing all household size data were excluded from the analysis.

^d^Past 3-year orphanhood percentages were calculated using lifetime orphanhood as the denominator.

### Intervention Exposure and Engagement

#### Exposure

Among the 614 participants who successfully installed the app, 600 (97.7%) engaged with at least some of the module content throughout the study, while 14 (2.3%) did not access any content. On average, participants visited the app 3.69 (SD 4.73) times, where a visit was defined as either the initial app launch or visiting a page after a 30-minute interval since the last page view. Each visit involved approximately 20.75 (SD 17.10) actions or page views, totaling 63.81 (SD 85.54) actions on average throughout the study.

For the guidance factor, 53.7% (330/614) of the participants were in self-guided clusters, while 46.2% (284/614) were in clusters allocated to WhatsApp groups. WhatsApp groups averaged 35.5 (SD 6.03) caregivers per group. Half (4/8, 50%) of the WhatsApp groups experienced delayed initiation due to rolling recruitment and the December 2022 Christmas break, with 2 groups postponed until January 2023. These delays likely contributed to participant attrition from the groups. Facilitators reported moderate WhatsApp message participation, but live-chat engagement was low, with attendance ranging from 1 to 16 participants per session (mean 2, SD 2.65).

For the app design factor, 54.1% (332/614) of the participants were in clusters allocated to the structured design and 45.9% (282/614) were in clusters allocated to the unstructured design. Research assistants activated the assigned design on participants’ phones based on their respective cluster. In 4 cases, the structured design was mistakenly activated; 3 of these participants did not respond to follow-ups and were treated as randomized in the analyses.

For the digital support factor, clusters assigned to basic support included 48% (295/614) of the participants, and enhanced support 52% (319/614). Given that participation was optional, attendance was not systematically recorded. Research assistants and fieldwork coordinators estimated that 67.1% (214/319) of the participants in enhanced support clusters attended training sessions. Nonattendance was primarily attributed to scheduling conflicts. Session duration varied, lasting anywhere from 5 to 20 minutes, depending on the group size and the number of questions asked.

#### Engagement

Table 4 summarizes the raw mean comparison of engagement outcomes for the entire sample and each factor level. Module completion rates among the 614 participants who successfully installed ParentApp were as follows: 38.4% (n=236) completed at least 25% (3/12) of the modules, 21.5% (n=132) completed at least 50% (6/12) of the modules, 13.8% (n=85) completed at least 75% (9/12) of the modules, and 8% (n=49) completed 100% (12/12) of the modules. Among the 600 participants who engaged with some module content, the average content completion rate across all 12 modules was 35.57% (SD 33.10%). This rate varied by factor levels: *guidance* (self-guided: mean 32.8%, SD 31.34% vs guided: mean 38.7%, SD 34.79%), *app design* (structured: mean 34.6%, SD 31.73% vs unstructured: mean 36.7%, SD 34.65%), and *digital support* (basic: mean 34.3%, SD 32.48% vs enhanced: mean 36.8%, SD 33.69%). For participants who started at least 1 module, survival analysis estimated a median retention time of 31 days (95% CI 25-36) from enrollment to last synchronization with the cloud server.

**Table 4:** Mean comparison of primary and secondary engagement outcomes.

Outcome		Total	Factor A: guidance			Factor B: app design			Factor C: digital support	
			Self-guided (n=330)	Guided (n=284)	Structured (n=332)		Unstructured (n=282)	Basic (n=295)		Enhanced (n=319)
**Primary engagement outcomes, mean (SD)**										
	App launches^a^	17.00 (22.59)	15.87 (20.93)	20.44 (24.18)	20.62 (26.81)		14.91 (15.86)	17.82 (23.67)		18.13 (21.56)
	Modules completed^b^	3.40 (3.64)	3.09 (3.41)	3.77 (3.85)	2.91 (3.04)		3.99 (4.16)	3.26 (3.51)		3.54 (3.75)
	Activity reviews^c^	1.62 (3.02)	1.39 (2.75)	1.88 (3.30)	0.49 (1.33)		2.95 (3.82)	1.48 (2.90)		1.74 (3.13)
**Secondary engagement outcomes, mean (SD)**										
	Modules started^d^	4.57 (4.13)	4.22 (3.92)	4.99 (4.33)	4.34 (4.03)		4.84 (4.23)	4.43 (4.04)		4.71 (4.21)
	Time spent in the app (min)^e^	54.99 (52.48)	48.79 (43.33)	62.22 (60.77)	51.46 (51.54)		59.19 (53.37)	53.39 (48.88)		56.49 (55.71)
	Positive behavior logs^f^	38.96 (104.07)	30.70 (75.52)	48.56 (129.06)	32.35 (66.25)		46.74 (135.44)	32.67 (89.18)		44.78 (115.99)

^a^Total app launches during the intervention period.

^b^Number of completed modules (0 to 12).

^c^Number of home practice activities reviewed (0 to 10).

^d^Number of modules started (0 to 12).

^e^Total minutes spent in the app.

^f^Number of self-reported positive parenting and mental health–promoting behaviors logged.

### Factor Effects on Primary and Secondary Outcomes

The main effect results for primary and secondary outcomes by factor are provided in Table 5. Following adjustments for baseline characteristics, caregivers who received WhatsApp group guidance in combination with ParentApp demonstrated significantly higher levels of engagement compared to those who were self-guided. Guided caregivers launched the app 2.93 times more (95% CI 1.84-4.68; *P*<.001), completed 1.29 times more modules (95% CI 1.05-1.58; *P*=.02), started 1.20 times more modules (95% CI 1.02-1.42; *P*=.03), spent 1.45 times more time in the app (95% CI 1.39-1.51; *P*<.001), and logged positive behaviors 2.73 times more frequently (95% CI 2.07-3.60; *P*<.001) than self-guided participants. Although guided caregivers initiated more home practice activity reviews compared to those who were self-guided, this difference was not statically significant (MR 2.17, 95% CI 0.84-5.63; *P*=.11).

The unstructured app design significantly outperformed the structured app design across all but 1 engagement outcome. App launch frequency did not differ significantly between designs (MR 0.88, 95% CI 0.70-1.11; *P*=.28). However, caregivers using the unstructured design completed 1.49 times more modules (95% CI 1.26-1.76; *P*<.001), initiated 7.49 times more home practice activity reviews (95% CI 5.19-10.82; *P*<.001), started 1.27 times more modules (95% CI 1.06-1.52; *P*=.01), and spent 1.84 times more time in the app (95% CI 1.70-1.99; *P*<.001) compared to those using the structured design. Notably, users of the unstructured design also logged 55.68 times more positive behaviors (95% CI 16.47-188.27; *P*<.001) compared to those using the structured design. A sensitivity analysis was conducted to address potential biases arising from extreme values in the positive behavior logs outcome (range 0-1342), where 10.4% (64/614) of the sample logged values exceeding 80 (deemed excessive in the study context). Models were rerun with positive behavior logs capped at 80. The results remained consistent with the original analyses (Table S2 of Multimedia Appendix 3).

**Table 5:** Main effects of experimental factors on engagement: unadjusted and adjusted results (N=614).

Factor level and outcomes		Unadjusted		Adjusted^a^	
		MR^b^ (95% CI)	*P* value	MR (95% CI)	*P* value
**Guided versus self-guided**					
	App launches	2.90 (1.85-4.54)	<.001	2.93 (1.84-4.68)	<.001
	Modules completed	1.29 (1.06-1.57)	.01	1.29 (1.05-1.58)	.02
	Activity reviews	2.23 (0.89-5.59)	.09	2.17 (0.84-5.63)	.11
	Modules started	1.23 (1.04-1.46)	.02	1.20 (1.02-1.42)	.03
	Time spent in the app	1.48 (1.44-1.52)	<.001	1.45 (1.39-1.51)	<.001
	Positive behavior logs	2.65 (2.03-3.45)	<.001	2.73 (2.07-3.60)	<.001
**Unstructured versus structured**					
	App launches	0.87 (0.69-1.10)	.25	0.88 (0.70-1.11)	.28
	Modules completed	1.45 (1.23-1.72)	<.001	1.49 (1.26-1.76)	<.001
	Activity reviews	6.84 (4.73-9.73)	<.001	7.49 (5.19-10.82)	<.001
	Modules started	1.24 (1.02-1.51)	.03	1.27 (1.06-1.52)	.01
	Time spent in the app	1.72 (1.67-1.77)	<.001	1.84 (1.70-1.99)	<.001
	Positive behavior logs	53.48 (17.44-164.03)	<.001	55.68 (16.47-188.27)	<.001
**Enhanced versus basic**					
	App launches	1.02 (0.78-1.32)	.91	1.01 (0.79-1.27)	.97
	Modules completed	1.09 (0.88-1.36)	.42	1.09 (0.87-1.36)	.45
	Activity reviews	1.15 (0.41-3.24)	.79	1.15 (0.39-3.34)	.80
	Modules started	1.07 (0.88-1.29)	.51	1.06 (0.88-1.27)	.53
	Time spent in the app	1.07 (0.87-1.30)	.53	1.07 (0.87-1.30)	.53
	Positive behavior logs	1.39 (0.78-2.48)	.27	1.44 (0.82-2.54)	.21

^a^Adjusted models included the following covariates: caregiver age, gender, financial stress, food insecurity, parenting stress, caregiver depression, overall child maltreatment, and overall positive parenting. All covariates were sample-mean centered.

^b^MR: mean ratio: estimated effects calculated as MR = exp (2β), where β is the unstandardized regression coefficient derived from the effect-coded experimental factors.

For the digital support factor, although enhanced support showed higher average engagement levels than basic support, these differences were not statistically significant (app launches: MR 1.01, 95% CI 0.79-1.27; *P*=.97; modules completed: MR 1.09, 95% CI 0.87-1.36; *P*=.45; home practice activity reviews: MR 1.15, 95% CI 0.39-3.34; *P*=.80; modules started: MR 1.06, 95% CI 0.88-1.27; *P*=.53; time spent in the app: MR 1.07, 95% CI 0.87-1.30; *P*=.53; and positive behavior logs: MR 1.44, 95% CI 0.82-2.54; *P*=.21).

### Factor Interaction Effects on Primary and Secondary Outcomes

Table 6 shows the main and 2-way interaction effects. Results for each outcome were derived from the same fitted model, adjusted for the same sample-mean centered covariates as the main effects analyses.

Although none of the factors showed significant main effects on app launches, a significant interaction between guidance and enhanced digital support (MR 1.69, 95% CI 1.06-2.71; *P*=.04) indicated that their combined effect positively influenced each other’s impact on the number of times a participant launched the app. For modules completed, all 3 factors showed significant main effects. In addition, guidance and enhanced digital support showed a significant interaction (MR 1.17, 95% CI 1.07-1.28; *P*<.001), suggesting a mutual positive influence on the number of modules completed. In the model analyzing home practice activity reviews, guidance and app design factors had significant main effects, while digital support did not. A significant interaction between guidance and the unstructured app design (MR 0.77, 95% CI 0.63-0.92; *P*=.004) indicated a decrease in activity reviews, while a significant interaction between guidance and enhanced digital support (MR 1.38, 95% CI 1.21-1.58; *P*<.001) indicated an increase in activity review rates.

**Table 6:** Interaction effects of experimental factors on engagement: adjusted results (N=614).

Outcome	Main effect							Interaction effect					
	Factor A^a^		Factor B^b^		Factor C^c^		A×B			A×C		B×C	
	MR^d^ (95% CI)	*P* value	MR (95% CI)	*P* value	MR (95% CI)	*P* value	MR (95% CI)		*P* value	MR (95% CI)	*P* value	MR (95% CI)	*P* value
App launches	1.53 (0.96-2.44)	.09	0.96 (0.54-1.71)	.89	1.01 (0.74-1.37)	.94	0.96 (0.72-1.26)		.75	1.69 (1.06-2.71)	.04	0.84 (0.47-1.50)	.56
Modules completed	1.22 (1.12-1.33)	<.001	1.42 (1.30-1.56)	<.001	1.09 (1.00-1.19)	.049	1.02 (0.94-1.12)		.59	1.17 (1.07-1.28)	<.001	1.09 (1.00-1.19)	.05
Activity reviews	1.63 (1.36-1.96)	<.001	7.06 (5.87-8.48)	<.001	1.15 (0.97-1.37)	.11	0.77 (0.64-0.92)		.004	1.38 (1.21-1.58)	<.001	1.07 (0.90-1.28)	.43
Modules started	1.17 (1.05-1.30)	.004	1.20 (1.07-1.34)	.001	1.07 (0.96-1.19)	.23	0.97 (0.88-1.08)		.63	1.24 (1.11-1.38)	<.001	1.10 (0.99-1.22)	.09
Time spent in the app	1.59 (0.97-2.62)	.07	2.08 (1.37-3.16)	.001	1.07 (0.56-2.03)	.85	1.48 (0.98-2.24)		.06	1.36 (0.82-2.23)	.23	0.36 (0.31-0.42)	<.001
Positive behavior logs	1.32 (0.58-2.99)	.51	2.37 (1.13-4.98)	.02	1.10 (0.41-2.96)	.85	1.10 (0.55-2.20)		.78	1.13 (0.50-2.58)	.77	1.24 (0.58-2.65)	.58

^a^WhatsApp group guided versus self-guided.

^b^Unstructured app design versus structured app design.

^c^Enhanced digital support versus basic digital support.

^d^MR: mean ratio: estimated effects calculated as MR = exp (2β), where β is the unstandardized regression coefficients derived from the effect-coded experimental factors.

Among secondary outcomes, the joint influence of guidance and enhanced digital support on modules started was significant (MR 1.24, 95% CI 1.11-1.38; *P*<.001), despite the digital support factor showing no significant main effect. Similarly, the unstructured design and enhanced digital support showed significant interaction effects for the time spent in the app, despite neither factor individually showing significance. However, their combined effects were attenuated (MR 0.36, 95% CI 0.31-0.42; *P*<.001), suggesting that their synergy led to a reduction in participants’ app use time.

## Discussion

### Principal Findings

This is the first known factorial trial of a digital parenting intervention for families with adolescents in an LMIC. It follows MOST principles [54] to optimize engagement with an app targeting socioeconomically disadvantaged families in Tanzania. Findings offer important insights into engagement-enhancing intervention design and implementation strategies for digital parenting interventions tailored to the unique contexts and needs of LMIC settings.

Receiving guidance through moderated WhatsApp groups significantly increased participant engagement across a wide range of engagement metrics. Compared to self-guided participants, those in WhatsApp groups launched the app more frequently, started and completed more program modules, spent more time in the app, and logged more positive parenting and mental health behaviors. These findings align with meta-analytic research on mental health apps and the broader digital health literature, which consistently shows that interventions incorporating some form of human guidance yield higher engagement rates compared to self-guided interventions [44,46,48-50]. However, our study advances this literature in 2 distinct ways. First, we directly compared self-guided and guided conditions within the same trial, whereas previous research has largely relied on cross-study comparisons [48-50]. Second, we implemented a group-based guidance model rather than individual approaches (eg, one-on-one phone consultations, videoconferencing, or email) commonly used in previous studies [26]. This shift toward a less resource-intensive delivery model enhances potential for future scalability.

The mechanisms through which human guidance facilitates engagement with digital interventions are not fully understood. One possible explanation is the role of the relationship quality between facilitators and participants [75]. Alternatively, guided participants may feel an increased sense of accountability in the presence of a facilitator [76]. The group-based format may also be important, as it enables interaction among caregivers. For instance, this setting allows participants with shared experiences to offer emotional and informational support [77] potentially enhancing engagement through social connection and community building. Indeed, a recent systematic review on peer-based features in digital interventions highlighted the importance of asynchronous communication features in supporting program engagement [51]. In addition to peer support benefits, findings from a systematic review of digital mental health interventions suggest that peer-to-peer groups are particularly effective in promoting engagement and retention when moderated by trained professionals [78]. Nevertheless, contact with facilitators or fellow participants may not be universally desirable or beneficial, as evidenced by participants who left WhatsApp groups prematurely in this study. These findings align with a systematic review showing that synchronous communication features, such as online video calls or chat rooms, were associated with reduced engagement [51]. Understanding which participants benefit from group-based support remains a critical direction for future research.

The unstructured app design, which featured flexible content access and culturally adapted, humanlike illustrations, significantly increased engagement compared to the structured design. Participants using the unstructured design completed more program modules, initiated more home practice reviews, started more modules, spent more time in the app, and logged more positive behaviors compared to those using the structured design. These findings align with research showing that user experience design, including app navigation and appearance, influences motivation and engagement with digital interventions [45,63]. However, as this study evaluated 2 app design packages rather than distinct components, it remains unclear which specific aspect of the unstructured design contributed to enhanced engagement. Future research would benefit from more granular testing and analysis of these design components to determine their individual impact. Enhanced engagement might be attributed to the flexible content progression, which allowed participants to move through the program at their preferred pace. Alternatively, increased engagement could reflect the greater relatability of culturally adapted images, which may have improved app acceptability among caregivers [26] and consequently boosted motivation to engage. While developing a universal app with content tailored for low-income contexts aimed to enhance scalability and reduce adaptation costs, our findings suggest that scalability alone does not ensure acceptability. Culturally adapted visual elements may therefore be crucial in fostering engagement with digital interventions.

A notable finding was the significant increase in home practice activity reviews among participants using the unstructured design. While the reason for this increase remains unclear, it could be attributed to the modular format of the design, which allowed participants to access home practice reviews without waiting for the 7-day cycle to be completed. Given that home practice of program skills is a key mechanism linking program implementation to behavioral outcomes in parenting interventions [79], including home practice engagement as an outcome is a study strength. However, the study does not establish a direct link between engaging in home practice reviews within the app and the actual application of these skills at home. Furthermore, this study only measured when participants initiated home practice reviews in the app, without capturing related measures such as self-reports of activity completion.

A surprising finding was the limited effectiveness of the digital support factor on engagement, which tended to have MRs close to 1 across most engagement outcomes. This suggests that augmenting app-specific assistance with general smartphone support may not yield additional benefits for participants. Unlike recent systematic reviews which assessed baseline digital literacy and its association with engagement [44,48], our study experimentally examined the provision of preprogram digital support. This was an important implementation consideration given our target population’s relatively low exposure to digital technology. While systematic reviews indicate that app literacy, defined as technological competency in using a smartphone app, is important for both uptake and engagement [46,80], it is plausible that the basic digital support module in our study adequately contributed to participants’ competency and perceived digital skills. Another potential reason could be that the enhanced digital training may have inadvertently reduced ParentApp engagement by introducing participants to competing apps and features, particularly among those with limited previous digital experience.

The impact of digital support on engagement might also have been influenced by various individual factors. The relatively young age of the sample (mean 35.94, SD 11.84 years) and their previous smartphone ownership may suggest sufficient baseline digital literacy. Moderator analyses could reveal whether factors such as caregiver age and other demographic characteristics influenced the results. If digital support proves effective only for specific participants, tailoring the type and intensity of support to individuals would be advantageous. Furthermore, it is essential to highlight that both digital support trainings were optional. The fieldwork team reported that while attendance was high for the basic training, some participants allocated to the enhanced training left the onboarding session early to resume economic and caregiving responsibilities. This reduced exposure could have attenuated the effects of the enhanced training; consideration is thus warranted when interpreting the results.

Interaction analyses found evidence that combining enhanced digital support with guidance led to increased engagement across 4 of the 6 measured outcomes. These interaction effects suggest the importance of smartphone literacy in promoting engagement, although enhanced digital support training alone may not be sufficient to sustain engagement. The combination of enhanced digital support and the unstructured design appeared to reduce app use time, possibly because increased navigation confidence paired with a simplified design allowed more efficient use. Further research is needed to help explain these findings.

### Limitations

Several limitations should be considered when interpreting the results. First, as this trial focused on engagement outcomes, we did not assess how the design and implementation factors influenced caregiver or adolescent outcomes. However, this trial serves as the optimization phase of the MOST framework within the broader ParentApp study, laying the groundwork for a comprehensive evaluation of the app’s effectiveness in the forthcoming randomized controlled trial [81]. Second, while the study’s focus on broader implementation strategies provides valuable insights for implementers and researchers, the generalizability of results to different LMIC settings or populations remains unclear. Further investigation into the factors influencing uptake and engagement across diverse behavioral domains, digital platforms, and delivery contexts is needed.

Third, data collection via automated tracking required internet access for synchronization with the cloud servers. As a result, participant engagement data may have been incomplete for those whose 1 GB monthly internet bundles depleted before data synchronization occurred, potentially leading to an underestimation of engagement rates. Efforts to address this included sending participants an SMS text message reminder toward the end of the study, along with an additional data bundle. However, it remains unclear to what extent these measures resolved the issue. Fourth, technical challenges hindered app installation and use. Despite enrolling 680 caregivers, surpassing the desired sample size of 640, installation difficulties affected 66 (9.7%) participants, and 14 (2.1%) did not to access any core intervention content after installation. These challenges stemmed primarily from internet and phone-to-app compatibility issues. Forthcoming qualitative research conducted in parallel to this study highlighted additional barriers to uptake and retention, including the selling of phones due to financial needs, deleting the app due to storage constraints, and program dropout due to severely damaged screens. Despite these challenges, overall content completion rates for participants who started at least 1 module was 35.57% of the program. While only 8% (49/614) of participants completed all 12 modules, completion rates were consistent with other digital parenting intervention studies in HICs, such as the 7.5% completion rate found in a large-scale study of the web-based ParentWorks program in Australia [39]. Nonetheless, investigating patterns of early dropout and identifying opportunities for keeping caregivers engaged throughout program delivery remain crucial areas for investigation.

### Strengths

This study has several notable strengths. A key strength is the application of a randomized factorial experiment to identify the combination of candidate factor levels that, while remaining feasible and acceptable, most effectively promoted engagement with ParentApp. Findings from this experiment played a pivotal role in selecting which factor levels to include in the forthcoming randomized controlled trial in Tanzania [81]. Following MOST framework decision-making guidelines [82], significant main effects guided the selection of effective factor levels, while lower-dose factor levels were retained when effects were null. Cost-effectiveness analysis and qualitative feedback then helped assess feasibility and acceptability of retained factor levels for the optimized intervention package. In addition, the findings from this experiment have contributed to the development and refinement of other digital parenting interventions, including in South Africa [83] and Malaysia [84].

Another key strength is the study context. Recruitment through local gatekeepers and community leaders yielded a large community sample in an LMIC. This approach aligns with how the intervention could be disseminated in the future and enhances the sustainability of the program. The sample also included 33.4% (205/614) men, a demographic typically underrepresented in parenting interventions [74]. This high proportion of male caregivers not only extends the generalizability of the results but also represents significant strides toward understanding how to engage underrepresented groups in digital parenting interventions in LMIC contexts. A forthcoming subgroup analysis of engagement predictors will provide further insights into this aspect of engagement.

Finally, unlike many digital intervention trials that provided participants with digital devices, this study’s sample consisted of participants who used their own smartphones, including older and low-cost models. This approach demonstrates the feasibility of implementing digital interventions in real-world, low-income settings and highlights the potential for cost-effective scale-up. Furthermore, by including participants with varying levels of technological access and proficiency, this study establishes a model for inclusive digital intervention design, particularly relevant in regions such as Tanzania, where smartphone access is expanding but still limited [85].

### Conclusions

While digital interventions hold immense promise for addressing health and service needs in LMICs, their effectiveness critically depends on ensuring meaningful engagement. This trial is the first of its kind to use a randomized factorial experiment to optimize participant engagement with an app-based parenting intervention in an LMIC. Moreover, this study adds to the paucity of research addressing the need for effective and accessible violence prevention interventions for adolescents in LMICs. While further investigation is needed to understand the impact of experimental factors on parenting and adolescent outcomes, our findings demonstrate that facilitator-moderated WhatsApp group guidance; an unstructured, culturally adapted app design; and preprogram digital support can enhance engagement with app-based parenting interventions. Continued exploration and refinement of these approaches is needed for developing evidence-based, scalable digital interventions capable of addressing parenting challenges and fostering positive outcomes for families in resource-constrained contexts.

## Appendix

Multimedia Appendix 1 TIDieR (Template for Intervention Description and Replication) checklist.

Multimedia Appendix 2 TIDieR (Template for Intervention Description and Replication) description.

Multimedia Appendix 3 Supplementary analyses.

Multimedia Appendix 4 Experimental conditions with effect coding.

Multimedia Appendix 5 CONSORT-eHEALTH checklist (V 1.6.1).

## Abbreviations

CONSORT: Consolidated Standards of Reporting Trials
CONSORT-EHEALTH: Consolidated Standards of Reporting Trials of Electronic and Mobile Health Applications and Online Telehealth
HIC: high-income country
ICS: Investing in Children and Strengthening their Societies
LMIC: low- and middle-income country
MOST: Multiphase Optimization Strategy
MR: mean ratio
NGO: nongovernmental organization
NIMR: National Institute for Medical Research
PLH: Parenting for Lifelong Health
TIDieR: Template for Intervention Description and Replication
VAC: violence against children

## Data Availability

The datasets generated or analyzed during this study are not currently publicly available due to ongoing analyses but will be made available via the Open Science Framework upon study completion. In the interim, datasets are available from the corresponding author on reasonable request.
