# Supplementary material for: Optimizing Engagement With a Smartphone App to Prevent Violence Against Adolescents: Results From a Cluster Randomized Factorial Trial in Tanzania
Source: J Med Internet Res. 2025 Mar 10;27:e60102. doi: 10.2196/60102 (PMC11933756; doi:10.2196/60102)
Supplement: Multimedia Appendix 2 [file jmir_v27i1e60102_app2.pdf]

Multimedia Appendix 2

Table S1. TIDieR description of ParentApp.

Item		Description
1.	Brief name	ParentApp
2.	Why	ParentApp is a smartphone app adapted from the in-person, group-based PLH Teens program, which was originally developed in South Africa to address the need for cost-effective, culturally appropriate violence prevention [1]. Initial evaluations of PLH Teens in South Africa demonstrated significant effects, including reduced child maltreatment, improved positive parenting practices, and decreased parental depression, stress, substance use, and financial stress [1]. PLH Teens has since been adapted and widely implemented across more than 18 LMICs [2], including a large-scale delivery in Tanzania to over 75,000 caregivers and adolescents [3]. Despite strong, emerging evidence for the effectiveness of the in-person program, the app-based delivery was adapted to address access and scalability challenges, including logistic barriers, human resource constraints, and economic costs associated with in-person delivery.
3.	What (materials)	The content material mirrors the content of in-person program in a condensed format and draws on existing evidence-based violence prevention interventions. In response to piloting and requests from families, adaptations were made to the program content to include (1) content to support caregiver mental health through mindfulness techniques, (2) content for families experiencing bereavement, (3) enhanced evidence-based content to prevent and respond to risks of sexual and online violence including No Means No Worldwide program content.
4.	What (procedures)	Eligible caregivers attended an in-person orientation and onboarding session where locally recruited trained research assistants and facilitators guided enrolled participants in downloading the app from Google Play and installing it on their smartphones. Once installed, caregivers participated in an optional app orientation training provided by facilitators and research assistants. ParentApp content focuses on relationship-building between caregivers and adolescents, stress management and problem-solving skills, non-violent approaches to discipline, and adolescent safety. This material is provided through text, images, and audio in 12 modules. A range of recurring activities are used in each module to reinforce learning and practical skills-building. These include:<br>**Mindfulness-based stress reduction activities**: Modules start with a “How are you?” emotional check-in, prompting users to assess their current

		state, followed by tailored feedback and relaxation exercises from a library of over 30 mindfulness activities. These promote presence, body awareness, and emotional regulation.<br>**Comics and scenario exploration**: Modules present parent-child scenarios through ‘negative comics’ and their positive alternatives. Users explore these through interactive segments such as “Question Time,” which includes tapping on emotions and selecting multiple-choice answers to understand different perspectives. This helps parents recognize potential challenges and learn how new skills can lead to positive outcomes.<br>**Reflection on benefits**: Short introductory messages and audio testimonials from other parents (translated into Swahili) underscore the benefits of each new skill, encouraging users to see their relevance and effectiveness.<br>**Essential Tools**: Modules include clear, concise overviews of key messages titled “Essential Tools.” These summaries facilitate the application of skills through simple headings, real-life examples, and links to module activities.<br>**Home practice activities**: Each module concludes with a “Ready to Practise?” activity, inviting users to plan how to apply the skill in their home environment. This section includes a testimonial from a local teenager to highlight the practical impact of the skill in a familiar context.<br>**Home activity reflection**: After completing home practice activities, a prompt (“How did your practice go?”) appears in the module overview. Users reflect on their experiences through multiple-choice questions and receive tailored solutions for overcoming any challenges they report. Positive feedback prompts reinforcement, while challenges trigger empathetic responses that acknowledge difficulties and provide practical guidance.<br>**ParentPoints**: Users can track their progress over time by collecting ParentPoints through self-reported use of positive parenting and mental health promoting practices which are aligned with module content: self-care practices ("relax," "treat yourself well," "praise yourself"), relationship-building behaviors ("one-on-one time," "praise your teen"), effective communication ("positive instructions"), emotion regulation ("respond calmly"), family management ("good money choices," "calm consequences"), and safety promotion ("keep your teen safe").
5.	Who (provides)	The intervention is delivered remotely as a smartphone app compatible with Android devices running version 5.5.1 or later. As part of the factorial design of the ParentApp optimisation study, local trained facilitators provided moderated WhatsApp groups support to half of the participants.

		Facilitators were paraprofessionals with prior experience supporting caregivers remotely during the Tanzanian pilot and were trained by CWBSA, an NGO with experience leading PLH training globally.
6.	How	The intervention was delivered remotely via an app. Half of the participants additionally received facilitator-moderated WhatsApp group guidance.
7.	When and how much (mode of delivery)	ParentApp material is delivered through text, images, and audio in 12 sessions. To encourage continued usage, three sets of push notifications are sent to participants: one scheduled within the first day of the user's initial app launch, another scheduled 6 days after the user's last app launch, and a third scheduled 30 days after the user's last app launch. For the WhatsApp groups, facilitators monitored caregiver discussions, shared helpful tips and reminders, addressed questions raised by participants, and led weekly 1-hour live chat sessions to encourage discussion and reflection.
8.	Where	The intervention was delivered via app to caregivers of adolescents aged 10-17 years in urban and peri-urban communities in the Mwanza region of Tanzania.
9.	Tailoring	Content is tailored according to whether participants selected using ParentApp alone or in a group setting with family or friends. Push notifications to encourage continued usage were tailored according to participant engagement.
10.	Modifications	Revisions and modifications for future iterations of delivery will be based on the findings of the current paper and from findings from qualitative focus group discussions covered in a subsequent publication.
11.	How well (planned)	Fidelity to the program was assessed based on engagement data (see results section of the paper) and via qualitative focus group (the results of which will be covered in a subsequent publication).
12.	How well (actual)	Automatically tracked engagement data assessing the actual adherence is reported in the present paper, and further information on adherence based on findings from qualitative focus group discussions with users will be covered in a subsequent publication.

Note. TIDieR: Template for Intervention Description and Replication

References

1. Cluver L, Meinck F, Steinert JI, et al. Parenting for Lifelong Health: A pragmatic cluster randomised controlled trial of a non-commercialised parenting programme for adolescents and their families in South Africa. *BMJ Global Health*. 2018;3(1):e000539. doi:10.1136/bmjgh-2017-000539

2. Shenderovich Y, Ward CL, Lachman JM, et al. Evaluating the dissemination and scale-up of two evidence-based parenting interventions to reduce violence against children: Study protocol. *Implementation Science Communications*. 2020;1(1):109. doi:10.1186/s43058-020-00086-6

3. Lachman J, Wamoyi J, Martin M, et al. Reducing family and school-based violence at scale in Tanzania: Results from a large-scale pre-post study of the Furaha Teens parenting programme. 2024;doi:10.31219/osf.io/7u8ct
