# Supplementary material for: Optimizing Engagement With a Smartphone App to Prevent Violence Against Adolescents: Results From a Cluster Randomized Factorial Trial in Tanzania
Source: J Med Internet Res. 2025 Mar 10;27:e60102. doi: 10.2196/60102 (PMC11933756; doi:10.2196/60102)
Supplement: Multimedia Appendix 3 [file jmir_v27i1e60102_app3.pdf]

Multimedia Appendix 3

Table S1. Multilevel logistic regression predicting app installation status as a function of randomization to experimental factors.

Outcome	Factor level[a]	MR[b]	95% CI	*P* value
App installed vs. app not installed	Guided vs. Self-guided	0.65	(0.38–1.12)	.12
	Unstructured vs. Structured	1.45	(0.82–2.56)	.21
	Enhanced vs. Basic	077	(0.45–1.33)	.34
**Random Effects**				
σ^2	3.29			
τ_{00} cluster	0.02			
ICC	0.01			
N	16			

[a]Guided: WhatsApp group guided versus self-guided; Unstructured: Unstructured app design versus structured app design; Enhanced: Enhanced digital support versus basic digital support.

[b]MR: mean ratio: estimated effects calculated as MR = exp(2β), where β is the unstandardized regression coefficient derived from the effect-coded experimental factors.

Observations = 680. Marginal R^2 / Conditional R^2 = 0.030 / 0.035

Table S2. Positive parenting and mental health behavior logs: sensitivity analysis.

		Unadjusted			Adjusted[c]		
Outcome	Factor level[b]	MR	95% CI	*P* value	MR	95% CI	*P* value
Positive behavior logs[a]	Guided vs. Self-guided	1.86	(1.46–2.36)	<.001	1.77	(1.39–2.24)	<.001
	Unstructured vs. Structured	16.22	(5.87–44.82)	<.001	16.92	(5.89–48.59)	<.001
	Enhanced vs. Basic	1.19	(0.83–1.72)	.34	1.20	(0.84–1.71)	.31

[a]Positive behavior logs capped at 80. where MR = exp(2 * β), with β representing the unstandardized regression coefficient.

[b]Guided: WhatsApp group guided versus self-guided; Unstructured: Unstructured app design versus structured app design; Enhanced: Enhanced digital support versus basic digital support.

[c]Adjusted for caregiver age, gender, and baseline scores: financial stress, food insecurity, parenting stress, caregiver depression, overall harsh discipline, overall positive parenting. All covariates were sample-mean centered.
