# Supplementary material for: Optimizing Engagement With a Smartphone App to Prevent Violence Against Adolescents: Results From a Cluster Randomized Factorial Trial in Tanzania
Source: J Med Internet Res. 2025 Mar 10;27:e60102. doi: 10.2196/60102 (PMC11933756; doi:10.2196/60102)
Supplement: Multimedia Appendix 4 [file jmir_v27i1e60102_app4.pdf]

**Multimedia Appendix 4**

**Table S1.** The 8 experimental conditions along with effect coding.

| Condition | Factor A: Guidance | Value | Factor B: App Design | Value | Factor C: Digital Support | Value |
|---|---|---|---|---|---|---|
| 1 | Self-guided | -1 | Unstructured | 1 | Enhanced | 1 |
| 2 | Self-guided | -1 | Unstructured | 1 | Basic | -1 |
| 3 | Self-guided | -1 | Structured | -1 | Enhanced | 1 |
| 4 | Self-guided | -1 | Structured | -1 | Basic | -1 |
| 5 | Guided | 1 | Unstructured | 1 | Enhanced | 1 |
| 6 | Guided | 1 | Unstructured | 1 | Basic | -1 |
| 7 | Guided | 1 | Structured | -1 | Enhanced | 1 |
| 8 | Guided | 1 | Structured | -1 | Basic | -1 |
