# Supplementary material for: Optimizing Engagement With a Smartphone App to Prevent Violence Against Adolescents: Results From a Cluster Randomized Factorial Trial in Tanzania
Source: J Med Internet Res. 2025 Mar 10;27:e60102. doi: 10.2196/60102 (PMC11933756; doi:10.2196/60102)
Supplement: Multimedia Appendix 5 [file jmir_v27i1e60102_app5.pdf]

CONSORT-EHEALTH (V 1.6.1) - Submission/Publication Form

The CONSORT-EHEALTH checklist is intended for authors of randomized trials evaluating web-based and Internet-based applications/interventions, including mobile interventions, electronic games (incl multiplayer games), social media, certain telehealth applications, and other interactive and/or networked electronic applications. Some of the items (e.g. all subitems under item 5 - description of the intervention) may also be applicable for other study designs.

The goal of the CONSORT EHEALTH checklist and guideline is to be
a) a guide for reporting for authors of RCTs,
b) to form a basis for appraisal of an ehealth trial (in terms of validity)

CONSORT-EHEALTH items/subitems are MANDATORY reporting items for studies published in the Journal of Medical Internet Research and other journals / scientific societies endorsing the checklist.

Items numbered 1., 2., 3., 4a., 4b etc are original CONSORT or CONSORT-NPT (non-pharmacologic treatment) items.
Items with Roman numerals (i., ii, iii, iv etc.) are CONSORT-EHEALTH extensions/clarifications.

As the CONSORT-EHEALTH checklist is still considered in a formative stage, we would ask that you also RATE ON A SCALE OF 1-5 how important/useful you feel each item is FOR THE PURPOSE OF THE CHECKLIST and reporting guideline (optional).

Mandatory reporting items are marked with a red *.
In the textboxes, either copy & paste the relevant sections from your manuscript into this form - please include any quotes from your manuscript in QUOTATION MARKS,
or answer directly by providing additional information not in the manuscript, or elaborating on why the item was not relevant for this study.

YOUR ANSWERS WILL BE PUBLISHED AS A SUPPLEMENTARY FILE TO YOUR PUBLICATION IN JMIR AND ARE CONSIDERED PART OF YOUR PUBLICATION (IF ACCEPTED).
Please fill in these questions diligently. Information will not be copyedited, so please use proper spelling and grammar, use correct capitalization, and avoid abbreviations.

DO NOT FORGET TO SAVE AS PDF _AND_ CLICK THE SUBMIT BUTTON SO YOUR ANSWERS ARE IN OUR DATABASE !!!

Citation Suggestion (if you append the pdf as Appendix we suggest to cite this paper in the caption):
Eysenbach G, CONSORT-EHEALTH Group
CONSORT-EHEALTH: Improving and Standardizing Evaluation Reports of Web-based and Mobile Health Interventions
J Med Internet Res 2011;13(4):e126

URL: http://www.jmir.org/2011/4/e126/
doi: 10.2196/jmir.1923
PMID: 22209829

roselinde.janowski@gmail.com Switch account

Not shared

Draft not saved

* Indicates required question

Your name *

First Last

Roselinde Janowski

Primary Affiliation (short), City, Country *

University of Toronto, Toronto, Canada

University of Oxford

Your e-mail address *

abc@gmail.com

roselinde.janowski@spi.ox.ac.uk

Title of your manuscript *

Provide the (draft) title of your manuscript.

Optimizing Engagement with a Smartphone App to Prevent Violence Against Adolescents in Tanzania: Results From a Cluster Randomized Factorial Trial

Name of your App/Software/Intervention *

If there is a short and a long/alternate name, write the short name first and add the long name in brackets.

ParentApp

Evaluated Version (if any)

e.g. "V1", "Release 2017-03-01", "Version 2.0.27913"

v0.16.9

Language(s) *

What language is the intervention/app in? If multiple languages are available, separate by comma (e.g. "English, French")

English, Kiswahili

URL of your Intervention Website or App

e.g. a direct link to the mobile app on app in appstore (itunes, Google Play), or URL of the website. If the intervention is a DVD or hardware, you can also link to an Amazon page.

https://plh-tz-optimisation.web.app/

URL of an image/screenshot (optional)

Your answer

Accessibility *

Can an enduser access the intervention presently?

- ○ access is free and open
- ○ access only for special usergroups, not open
- ○ access is open to everyone, but requires payment/subscription/in-app purchases
- ○ app/intervention no longer accessible
- ◉ Other: Access is free and open, but only globally available via the temporary

Primary Medical Indication/Disease/Condition *

e.g. "Stress", "Diabetes", or define the target group in brackets after the condition, e.g. "Autism (Parents of children with)", "Alzheimers (Informal Caregivers of)"

Violence against adolescents (Caregivers of a

Primary Outcomes measured in trial *

comma-separated list of primary outcomes reported in the trial

es completed, home practice activities review

Secondary/other outcomes

Are there any other outcomes the intervention is expected to affect?

Engagement: time spent on app, modules started, and positive parenting and mental health habits logged

Recommended "Dose" *

What do the instructions for users say on how often the app should be used?

- ( ) Approximately Daily
- (x) Approximately Weekly
- ( ) Approximately Monthly
- ( ) Approximately Yearly
- ( ) "as needed"
- ( ) Other:

Approx. Percentage of Users (starters) still using the app as recommended after 3 months *

- (x) unknown / not evaluated
- ( ) 0-10%
- ( ) 11-20%
- ( ) 21-30%
- ( ) 31-40%
- ( ) 41-50%
- ( ) 51-60%
- ( ) 61-70%
- ( ) 71%-80%
- ( ) 81-90%
- ( ) 91-100%
- ( ) Other:

Overall, was the app/intervention effective? *

- ( ) yes: all primary outcomes were significantly better in intervention group vs control
- (x) partly: SOME primary outcomes were significantly better in intervention group vs control
- ( ) no statistically significant difference between control and intervention
- ( ) potentially harmful: control was significantly better than intervention in one or more outcomes
- ( ) inconclusive: more research is needed
- ( ) Other:

Article Preparation Status/Stage *

At which stage in your article preparation are you currently (at the time you fill in this form)

- ( ) not submitted yet - in early draft status
- (x) not submitted yet - in late draft status, just before submission
- ( ) submitted to a journal but not reviewed yet
- ( ) submitted to a journal and after receiving initial reviewer comments
- ( ) submitted to a journal and accepted, but not published yet
- ( ) published
- ( ) Other:

Journal *

If you already know where you will submit this paper (or if it is already submitted), please provide the journal name (if it is not JMIR, provide the journal name under "other")

- ( ) not submitted yet / unclear where I will submit this
- (•) Journal of Medical Internet Research (JMIR)
- ( ) JMIR mHealth and UHealth
- ( ) JMIR Serious Games
- ( ) JMIR Mental Health
- ( ) JMIR Public Health
- ( ) JMIR Formative Research
- ( ) Other JMIR sister journal
- ( ) Other:

Is this a full powered effectiveness trial or a pilot/feasibility trial? *

- ( ) Pilot/feasibility
- (•) Fully powered

Manuscript tracking number *

If this is a JMIR submission, please provide the manuscript tracking number under "other" (The ms tracking number can be found in the submission acknowledgement email, or when you login as author in JMIR. If the paper is already published in JMIR, then the ms tracking number is the four-digit number at the end of the DOI, to be found at the bottom of each published article in JMIR)

- (•) no ms number (yet) / not (yet) submitted to / published in JMIR
- ( ) Other:

TITLE AND ABSTRACT

1a) TITLE: Identification as a randomized trial in the title

1a) Does your paper address CONSORT item 1a? *

I.e does the title contain the phrase "Randomized Controlled Trial"? (if not, explain the reason under "other")

- (selected) yes
- ( ) Other:

1a-i) Identify the mode of delivery in the title

Identify the mode of delivery. Preferably use “web-based” and/or “mobile” and/or “electronic game” in the title. Avoid ambiguous terms like “online”, “virtual”, “interactive”. Use “Internet-based” only if Intervention includes non-web-based Internet components (e.g. email), use “computer-based” or “electronic” only if offline products are used. Use “virtual” only in the context of “virtual reality” (3-D worlds). Use “online” only in the context of “online support groups”. Complement or substitute product names with broader terms for the class of products (such as “mobile” or “smart phone” instead of “iphone”), especially if the application runs on different platforms.

	1	2	3	4	5	
subitem not at all important	○	○	○	○	◉	essential

Clear selection

Does your paper address subitem 1a-i? *

Copy and paste relevant sections from manuscript title (include quotes in quotation marks "like this" to indicate direct quotes from your manuscript), or elaborate on this item by providing additional information not in the ms, or briefly explain why the item is not applicable/relevant for your study

The term "Smartphone App" is used in the title.

1a-ii) Non-web-based components or important co-interventions in title

Mention non-web-based components or important co-interventions in title, if any (e.g., "with telephone support").

	1	2	3	4	5	
subitem not at all important	○	○	○	○	◉	essential

Clear selection

Does your paper address subitem 1a-ii?

Copy and paste relevant sections from manuscript title (include quotes in quotation marks "like this" to indicate direct quotes from your manuscript), or elaborate on this item by providing additional information not in the ms, or briefly explain why the item is not applicable/relevant for your study

The term "Factorial Trial" is used to denote that there were multiple treatment components used to optimize the smartphone app. Due to space constraints, the three factors and their levels were not included in the title but have been clearly defined in the abstract "guidance (self-guided/guided via facilitator-moderated WhatsApp groups), app design (structured/unstructured), and pre-program digital support (basic/enhanced)".

1a-iii) Primary condition or target group in the title

Mention primary condition or target group in the title, if any (e.g., "for children with Type I Diabetes") Example: A Web-based and Mobile Intervention with Telephone Support for Children with Type I Diabetes: Randomized Controlled Trial

	1	2	3	4	5	
subitem not at all important	○	○	○	○	◉	essential

Clear selection

Does your paper address subitem 1a-iii? *

Copy and paste relevant sections from manuscript title (include quotes in quotation marks "like this" to indicate direct quotes from your manuscript), or elaborate on this item by providing additional information not in the ms, or briefly explain why the item is not applicable/relevant for your study

The title reflects that the intervention is designed to prevent violence against adolescents: "Optimizing Engagement with a Smartphone App to Prevent Violence Against Adolescents in Tanzania..."

1b) ABSTRACT: Structured summary of trial design, methods, results, and conclusions

NPT extension: Description of experimental treatment, comparator, care providers, centers, and blinding status.

1b-i) Key features/functionalities/components of the intervention and comparator in the METHODS section of the ABSTRACT

Mention key features/functionalities/components of the intervention and comparator in the abstract. If possible, also mention theories and principles used for designing the site. Keep in mind the needs of systematic reviewers and indexers by including important synonyms. (Note: Only report in the abstract what the main paper is reporting. If this information is missing from the main body of text, consider adding it)

	1	2	3	4	5	
subitem not at all important	○	○	○	○	●	essential

Clear selection

Does your paper address subitem 1b-i? *

Copy and paste relevant sections from the manuscript abstract (include quotes in quotation marks "like this" to indicate direct quotes from your manuscript), or elaborate on this item by providing additional information not in the ms, or briefly explain why the item is not applicable/relevant for your study

The abstract identifies the specific methodological principles as well as the components of the intervention. See Methods section below:

Methods: "Following Multiphase Optimization Strategy (MOST) principles, we conducted a 2x2x2 cluster randomized factorial trial involving caregivers of adolescents aged 10-17 years"

"Each cluster was randomized to one of two levels of each factor: guidance (self-guided/guided via facilitator-moderated WhatsApp groups), app design (structured/unstructured), and pre-program digital support (basic/enhanced)"

1b-ii) Level of human involvement in the METHODS section of the ABSTRACT

Clarify the level of human involvement in the abstract, e.g., use phrases like "fully automated" vs. "therapist/nurse/care provider/physician-assisted" (mention number and expertise of providers involved, if any). (Note: Only report in the abstract what the main paper is reporting. If this information is missing from the main body of text, consider adding it)

	1	2	3	4	5	
subitem not at all important	○	○	○	○	◉	essential

Clear selection

Does your paper address subitem 1b-ii?

Copy and paste relevant sections from the manuscript abstract (include quotes in quotation marks "like this" to indicate direct quotes from your manuscript), or elaborate on this item by providing additional information not in the ms, or briefly explain why the item is not applicable/relevant for your study

The Methods section of the abstract clearly indicates the level of human involvement. See Methods section below:

"Each cluster was randomized to one of two levels of each factor: guidance (self-guided/guided via facilitator-moderated WhatsApp groups), app design (structured/unstructured), and pre-program digital support (basic/enhanced)"

1b-iii) Open vs. closed, web-based (self-assessment) vs. face-to-face assessments in the METHODS section of the ABSTRACT

Mention how participants were recruited (online vs. offline), e.g., from an open access website or from a clinic or a closed online user group (closed usergroup trial), and clarify if this was a purely web-based trial, or there were face-to-face components (as part of the intervention or for assessment). Clearly say if outcomes were self-assessed through questionnaires (as common in web-based trials). Note: In traditional offline trials, an open trial (open-label trial) is a type of clinical trial in which both the researchers and participants know which treatment is being administered. To avoid confusion, use “blinded” or “unblinded” to indicated the level of blinding instead of “open”, as “open” in web-based trials usually refers to “open access” (i.e. participants can self-enrol). (Note: Only report in the abstract what the main paper is reporting. If this information is missing from the main body of text, consider adding it)

	1	2	3	4	5	
subitem not at all important	○	○	○	◉	○	essential

Clear selection

Does your paper address subitem 1b-iii?

Copy and paste relevant sections from the manuscript abstract (include quotes in quotation marks "like this" to indicate direct quotes from your manuscript), or elaborate on this item by providing additional information not in the ms, or briefly explain why the item is not applicable/relevant for your study

The abstract addresses this in the Methods section:

"Caregivers were recruited by community representatives from 16 urban and peri-urban communities (i.e., clusters) in the Mwanza region of Tanzania"

"Primary outcomes were automatically tracked measures of engagement"

1b-iv) RESULTS section in abstract must contain use data

Report number of participants enrolled/assessed in each group, the use/uptake of the intervention (e.g., attrition/adherence metrics, use over time, number of logins etc.), in addition to primary/secondary outcomes. (Note: Only report in the abstract what the main paper is reporting. If this information is missing from the main body of text, consider adding it)

	1	2	3	4	5	
subitem not at all important	○	○	○	○	●	essential

Clear selection

Does your paper address subitem 1b-iv?

Copy and paste relevant sections from the manuscript abstract (include quotes in quotation marks "like this" to indicate direct quotes from your manuscript), or elaborate on this item by providing additional information not in the ms, or briefly explain why the item is not applicable/relevant for your study

User data is provided in the abstract as follows:

"...engagement data from 614 caregivers were analyzed, of which 33.4% were male. Compared to self-guided participants, receiving guidance alongside the app led to significantly more app launches (MR 2.93; 95% CI [1.84–4.68]), more modules completed (MR 1.29; 95% CI [1.05–1.58]), more time spent on the app (MR 1.45; 95% CI 1.39–1.51), more modules started (MR 1.20; 95% CI [1.02–1.42]), and more positive habits logged (MR 2.73; 95% CI [2.07–3.60]). Compared to using the structured design, unstructured design use resulted in significantly more modules completed (MR 1.49; 95% CI [1.26–1.76]), more home practice activities reviewed (MR 7.49; 95% CI [5.19–10.82]), more time spent on the app (MR 1.84; 95% CI [1.70–1.99]), more modules started (MR 1.27; 95% CI [1.06–1.52]), and more positive habits logged (MR 55.68; 95% CI [16.48–188.14]). While analyses did not detect an effect of enhanced digital support on directly observed engagement, the combination of enhanced digital support and guidance positively influenced engagement across a range of outcomes"

1b-v) CONCLUSIONS/DISCUSSION in abstract for negative trials

Conclusions/Discussions in abstract for negative trials: Discuss the primary outcome - if the trial is negative (primary outcome not changed), and the intervention was not used, discuss whether negative results are attributable to lack of uptake and discuss reasons. (Note: Only report in the abstract what the main paper is reporting. If this information is missing from the main body of text, consider adding it)

	1	2	3	4	5	
subitem not at all important	○	○	○	○	◉	essential

Clear selection

Does your paper address subitem 1b-v?

Copy and paste relevant sections from the manuscript abstract (include quotes in quotation marks "like this" to indicate direct quotes from your manuscript), or elaborate on this item by providing additional information not in the ms, or briefly explain why the item is not applicable/relevant for your study

This item is not relevant to the current paper.

INTRODUCTION

2a) In INTRODUCTION: Scientific background and explanation of rationale

2a-i) Problem and the type of system/solution

Describe the problem and the type of system/solution that is object of the study: intended as stand-alone intervention vs. incorporated in broader health care program? Intended for a particular patient population? Goals of the intervention, e.g., being more cost-effective to other interventions, replace or complement other solutions? (Note: Details about the intervention are provided in “Methods” under 5)

	1	2	3	4	5	
subitem not at all important	○	○	○	○	◉	essential

Clear selection

Does your paper address subitem 2a-i? *

Copy and paste relevant sections from the manuscript (include quotes in quotation marks "like this" to indicate direct quotes from your manuscript), or elaborate on this item by providing additional information not in the ms, or briefly explain why the item is not applicable/relevant for your study

The Introduction outlines the problem of interest: "Empirical understanding of optimal design and implementation strategies for engaging parents in digital interventions, particularly in LMIC contexts, remains limited"

"Notably, only one known study has experimentally tested intervention design components that may promote engagement with a digital parenting intervention [36], and none have been conducted in LMICs"

The goals of the study were: "...to evaluate the impact of three intervention design and implementation factors – guidance, app design, and digital support – on caregiver engagement with ParentApp, tailored for socioeconomically vulnerable caregivers of adolescents in Tanzania"

2a-ii) Scientific background, rationale: What is known about the (type of) system

Scientific background, rationale: What is known about the (type of) system that is the object of the study (be sure to discuss the use of similar systems for other conditions/diagnoses, if appropiate), motivation for the study, i.e. what are the reasons for and what is the context for this specific study, from which stakeholder viewpoint is the study performed, potential impact of findings [2]. Briefly justify the choice of the comparator.

	1	2	3	4	5	
subitem not at all important	○	○	○	○	◉	essential

Clear selection

Does your paper address subitem 2a-ii? *

Copy and paste relevant sections from the manuscript (include quotes in quotation marks "like this" to indicate direct quotes from your manuscript), or elaborate on this item by providing additional information not in the ms, or briefly explain why the item is not applicable/relevant for your study

The Introduction reviews relevant literature to digital parenting programs, "Emerging evidence from systematic reviews and meta-analyses conducted in high-income countries (HICs) demonstrate the potential effectiveness of digital parenting interventions [eg, 18,19-24]. However, these programs frequently face challenges of low engagement and high dropout rates [25]"

The introduction reviews relevant literature on the effects of design/implementation features on program engagement, "reviews of app-based health interventions have identified several design and delivery factors linked to increased engagement, including personalized content and feedback [37-39], user-friendliness [40], a visually appealing layout and the integration of a tutorial on how to use the app [37], human guidance [41-43], peer-based asynchronous communication features [44], and reminders [28,37,45]"

2b) In INTRODUCTION: Specific objectives or hypotheses

Does your paper address CONSORT subitem 2b? *

Copy and paste relevant sections from the manuscript (include quotes in quotation marks "like this" to indicate direct quotes from your manuscript), or elaborate on this item by providing additional information not in the ms, or briefly explain why the item is not applicable/relevant for your study

Yes, the introduction concludes by stating the specific objectives of the study, "The primary objective of this trial was to evaluate the effectiveness of these factors on primary and secondary engagement outcomes. An exploratory objective was to identify potential interaction effects between the factors."

METHODS

3a) Description of trial design (such as parallel, factorial) including allocation ratio

Does your paper address CONSORT subitem 3a? *

Copy and paste relevant sections from the manuscript (include quotes in quotation marks "like this" to indicate direct quotes from your manuscript), or elaborate on this item by providing additional information not in the ms, or briefly explain why the item is not applicable/relevant for your study

Yes, the trial design is is clearly stated, "The trial used a cluster randomized factorial design with three experimental factors: (A) guidance (self-guided/guided), (B) app design (structured/unstructured), and (C) digital support (basic/enhanced). Each factor was varied at two levels (lower-dose/inactive versus higher-dose/active) yielding an 8-condition (2x2x2) trial. A full factorial design was used, allowing all clusters to potentially be randomized to all combinations of factors and their corresponding levels"

3b) Important changes to methods after trial commencement (such as eligibility criteria), with reasons

Does your paper address CONSORT subitem 3b? *

Copy and paste relevant sections from the manuscript (include quotes in quotation marks "like this" to indicate direct quotes from your manuscript), or elaborate on this item by providing additional information not in the ms, or briefly explain why the item is not applicable/relevant for your study

Not applicable - we did not make any relevant changes to the study methods after enrollment began or during the period data was being collected for this study.

3b-i) Bug fixes, Downtimes, Content Changes

Bug fixes, Downtimes, Content Changes: ehealth systems are often dynamic systems. A description of changes to methods therefore also includes important changes made on the intervention or comparator during the trial (e.g., major bug fixes or changes in the functionality or content) (5-iii) and other “unexpected events” that may have influenced study design such as staff changes, system failures/downtimes, etc. [2].

	1	2	3	4	5	
subitem not at all important	○	○	○	○	◉	essential

Clear selection

Does your paper address subitem 3b-i?

Copy and paste relevant sections from the manuscript (include quotes in quotation marks "like this" to indicate direct quotes from your manuscript), or elaborate on this item by providing additional information not in the ms, or briefly explain why the item is not applicable/relevant for your study

Not applicable - we encountered no major down times, content changes, or bug fixes after launch of this trial.

4a) Eligibility criteria for participants

Does your paper address CONSORT subitem 4a? *

Copy and paste relevant sections from the manuscript (include quotes in quotation marks "like this" to indicate direct quotes from your manuscript), or elaborate on this item by providing additional information not in the ms, or briefly explain why the item is not applicable/relevant for your study

Yes, the paper states: "Eligible clusters were sub-wards that had not previously participated in the preliminary testing phases of the app and had a sufficient number of households (40 or more) with smartphone ownership. Participants within each cluster were primary caregivers of at least one adolescent aged 10 to 17 years. Eligible caregivers were required to be at least 18 years of age, live in the same household as their adolescent for at least 4 nights per week in the previous month, have regular access to an Android smartphone, and provide written informed consent. Participants were excluded from the study if they were unable to read or had a severe learning disability that affected their ability to provide informed consent."

4a-i) Computer / Internet literacy

Computer / Internet literacy is often an implicit “de facto” eligibility criterion - this should be explicitly clarified.

	1	2	3	4	5	
subitem not at all important	○	○	○	◉	○	essential

Clear selection

Does your paper address subitem 4a-i?

Copy and paste relevant sections from the manuscript (include quotes in quotation marks "like this" to indicate direct quotes from your manuscript), or elaborate on this item by providing additional information not in the ms, or briefly explain why the item is not applicable/relevant for your study

Participants were only eligible if they were literate and had regular access to a smartphone. The experimental factor "digital support" specifically focused on supporting participants, including those with limited digital literacy.

4a-ii) Open vs. closed, web-based vs. face-to-face assessments:

Open vs. closed, web-based vs. face-to-face assessments: Mention how participants were recruited (online vs. offline), e.g., from an open access website or from a clinic, and clarify if this was a purely web-based trial, or there were face-to-face components (as part of the intervention or for assessment), i.e., to what degree got the study team to know the participant. In online-only trials, clarify if participants were quasi-anonymous and whether having multiple identities was possible or whether technical or logistical measures (e.g., cookies, email confirmation, phone calls) were used to detect/prevent these.

	1	2	3	4	5	
subitem not at all important	○	○	○	○	◉	essential

Clear selection

Does your paper address subitem 4a-ii? *

Copy and paste relevant sections from the manuscript (include quotes in quotation marks "like this" to indicate direct quotes from your manuscript), or elaborate on this item by providing additional information not in the ms, or briefly explain why the item is not applicable/relevant for your study

The paper addresses these points, "Recruitment of caregivers by community representatives took place either face-to-face or via phone call."

4a-iii) Information giving during recruitment

Information given during recruitment. Specify how participants were briefed for recruitment and in the informed consent procedures (e.g., publish the informed consent documentation as appendix, see also item X26), as this information may have an effect on user self-selection, user expectation and may also bias results.

	1	2	3	4	5	
subitem not at all important	○	○	○	○	◉	essential

Clear selection

Does your paper address subitem 4a-iii?

Copy and paste relevant sections from the manuscript (include quotes in quotation marks "like this" to indicate direct quotes from your manuscript), or elaborate on this item by providing additional information not in the ms, or briefly explain why the item is not applicable/relevant for your study

The information sheet and consent form clearly laid out the study purpose, risks, benefits, and other required consent elements. This was published as a supplementary file with the published study protocol.

4b) Settings and locations where the data were collected

Does your paper address CONSORT subitem 4b? *

Copy and paste relevant sections from the manuscript (include quotes in quotation marks "like this" to indicate direct quotes from your manuscript), or elaborate on this item by providing additional information not in the ms, or briefly explain why the item is not applicable/relevant for your study

The setting and location is described as: "Target communities were urban and peri-urban sub-wards characterized by high rates of poverty in the Mwanza region, Tanzania."

4b-i) Report if outcomes were (self-)assessed through online questionnaires

Clearly report if outcomes were (self-)assessed through online questionnaires (as common in web-based trials) or otherwise.

	1	2	3	4	5	
subitem not at all important	○	○	○	○	◉	essential

Clear selection

Does your paper address subitem 4b-i? *

Copy and paste relevant sections from the manuscript (include quotes in quotation marks "like this" to indicate direct quotes from your manuscript), or elaborate on this item by providing additional information not in the ms, or briefly explain why the item is not applicable/relevant for your study

Yes, the paper states: "...participants self-administered a baseline assessment integrated within the app’s first module"

4b-ii) Report how institutional affiliations are displayed

Report how institutional affiliations are displayed to potential participants [on ehealth media], as affiliations with prestigious hospitals or universities may affect volunteer rates, use, and reactions with regards to an intervention.(Not a required item – describe only if this may bias results)

	1	2	3	4	5	
subitem not at all important	○	○	○	○	◉	essential

Clear selection

Does your paper address subitem 4b-ii?

Copy and paste relevant sections from the manuscript (include quotes in quotation marks "like this" to indicate direct quotes from your manuscript), or elaborate on this item by providing additional information not in the ms, or briefly explain why the item is not applicable/relevant for your study

Not applicable - the institutional affiliations were mentioned in the study information sheet and consent, and were only displayed in the Terms & Conditions and Privacy Policy of the app. They were not displayed in any other parts of the app.

5) The interventions for each group with sufficient details to allow replication, including how and when they were actually administered

5-i) Mention names, credential, affiliations of the developers, sponsors, and owners

Mention names, credential, affiliations of the developers, sponsors, and owners [6] (if authors/evaluators are owners or developer of the software, this needs to be declared in a "Conflict of interest" section or mentioned elsewhere in the manuscript).

	1	2	3	4	5	
subitem not at all important	○	○	○	○	◉	essential

Clear selection

Does your paper address subitem 5-i?

Copy and paste relevant sections from the manuscript (include quotes in quotation marks "like this" to indicate direct quotes from your manuscript), or elaborate on this item by providing additional information not in the ms, or briefly explain why the item is not applicable/relevant for your study

The software used in this study was jointly developed for the study by investigators, research partners, and implementing partners, as described in the paper, "The development and delivery of ParentApp in Tanzania were jointly led by the Universities of Oxford and Cape Town, IDEMS International, Tanzania's National Institute for Medical Research, Parenting for Lifelong Health, Clowns Without Borders South Africa, and Investing in Children and Strengthening their Societies, in collaboration with UNICEF, WHO, and the Tanzanian National Government "

The software is open-access and not for commercial use. The conflict of interest statement includes the following: "ParentApp was developed and implemented by members of the research team...No profit or financial gain will be made from implementation and dissemination of the intervention"

5-ii) Describe the history/development process

Describe the history/development process of the application and previous formative evaluations (e.g., focus groups, usability testing), as these will have an impact on adoption/use rates and help with interpreting results.

	1	2	3	4	5	
subitem not at all important	○	○	○	○	◉	essential

Clear selection

Does your paper address subitem 5-ii?

Copy and paste relevant sections from the manuscript (include quotes in quotation marks "like this" to indicate direct quotes from your manuscript), or elaborate on this item by providing additional information not in the ms, or briefly explain why the item is not applicable/relevant for your study

The development process is described in the paper as such, "The final design and implementation strategy evaluated in this trial was informed by: (1) multiphase adaptation and co-development with researchers, program specialists, and technical experts, as well as PLH for Teens participants and facilitators in South Africa; (2) user-testing interviews with 24 participants from 9 African countries [55], (3) an early mixed-methods feasibility pilot of the app delivered as a self-guided intervention with 107 caregivers in South Africa; and (4) a mixed-method pilot of the app delivered with remote guidance (phone calls or moderated WhatsApp groups) to 103 caregiver-adolescent dyads in rural and urban Tanzania"

5-iii) Revisions and updating

Revisions and updating. Clearly mention the date and/or version number of the application/intervention (and comparator, if applicable) evaluated, or describe whether the intervention underwent major changes during the evaluation process, or whether the development and/or content was “frozen” during the trial. Describe dynamic components such as news feeds or changing content which may have an impact on the replicability of the intervention (for unexpected events see item 3b).

	1	2	3	4	5	
subitem not at all important	○	○	○	○	◉	essential

Clear selection

Does your paper address subitem 5-iii?

Copy and paste relevant sections from the manuscript (include quotes in quotation marks "like this" to indicate direct quotes from your manuscript), or elaborate on this item by providing additional information not in the ms, or briefly explain why the item is not applicable/relevant for your study

There were no major changes to the intervention design or content after study launch

5-iv) Quality assurance methods

Provide information on quality assurance methods to ensure accuracy and quality of information provided [1], if applicable.

	1	2	3	4	5	
subitem not at all important	○	○	○	◉	○	essential

Clear selection

Does your paper address subitem 5-iv?

Copy and paste relevant sections from the manuscript (include quotes in quotation marks "like this" to indicate direct quotes from your manuscript), or elaborate on this item by providing additional information not in the ms, or briefly explain why the item is not applicable/relevant for your study

The paper states: "Data monitoring protocols were implemented to ensure accurate tracking and to verify that participants received the correct app version based on cluster allocation"

5-v) Ensure replicability by publishing the source code, and/or providing screenshots/screen-capture video, and/or providing flowcharts of the algorithms used

Ensure replicability by publishing the source code, and/or providing screenshots/screen-capture video, and/or providing flowcharts of the algorithms used. Replicability (i.e., other researchers should in principle be able to replicate the study) is a hallmark of scientific reporting.

	1	2	3	4	5	
subitem not at all important	○	○	○	○	◉	essential

Clear selection

Does your paper address subitem 5-v?

Copy and paste relevant sections from the manuscript (include quotes in quotation marks "like this" to indicate direct quotes from your manuscript), or elaborate on this item by providing additional information not in the ms, or briefly explain why the item is not applicable/relevant for your study

We provide a detailed overview of the intervention content and design in this paper, including a TIDieR checklist. We also include screenshots of the two app designs evaluated in the study to illustrate the layout and visual elements of the intervention. The source code is also available upon request.

5-vi) Digital preservation

Digital preservation: Provide the URL of the application, but as the intervention is likely to change or disappear over the course of the years; also make sure the intervention is archived (Internet Archive, webcitation.org, and/or publishing the source code or screenshots/videos alongside the article). As pages behind login screens cannot be archived, consider creating demo pages which are accessible without login.

	1	2	3	4	5	
subitem not at all important	○	○	◉	○	○	essential

Clear selection

Does your paper address subitem 5-vi?

Copy and paste relevant sections from the manuscript (include quotes in quotation marks "like this" to indicate direct quotes from your manuscript), or elaborate on this item by providing additional information not in the ms, or briefly explain why the item is not applicable/relevant for your study

A temporary demo web page is available which is assessable without login: https://plh-tz-optimisation.web.app/

5-vii) Access

Access: Describe how participants accessed the application, in what setting/context, if they had to pay (or were paid) or not, whether they had to be a member of specific group. If known, describe how participants obtained “access to the platform and Internet” [1]. To ensure access for editors/reviewers/readers, consider to provide a “backdoor” login account or demo mode for reviewers/readers to explore the application (also important for archiving purposes, see vi).

	1	2	3	4	5	
subitem not at all important	○	○	○	○	◉	essential

Clear selection

Does your paper address subitem 5-vii? *

Copy and paste relevant sections from the manuscript (include quotes in quotation marks "like this" to indicate direct quotes from your manuscript), or elaborate on this item by providing additional information not in the ms, or briefly explain why the item is not applicable/relevant for your study

The paper states: "at the in-person onboarding session... research assistants guided enrolled caregivers in downloading the app from Google Play and installing it on their smartphones".

No login details were required to gain access to the app.

5-viii) Mode of delivery, features/functionalities/components of the intervention and comparator, and the theoretical framework

Describe mode of delivery, features/functionalities/components of the intervention and comparator, and the theoretical framework [6] used to design them (instructional strategy [1], behaviour change techniques, persuasive features, etc., see e.g., [7, 8] for terminology). This includes an in-depth description of the content (including where it is coming from and who developed it) [1],” whether [and how] it is tailored to individual circumstances and allows users to track their progress and receive feedback” [6]. This also includes a description of communication delivery channels and – if computer-mediated communication is a component – whether communication was synchronous or asynchronous [6]. It also includes information on presentation strategies [1], including page design principles, average amount of text on pages, presence of hyperlinks to other resources, etc. [1].

	1	2	3	4	5	
subitem not at all important	○	○	○	○	◉	essential

Clear selection

Does your paper address subitem 5-viii? *

Copy and paste relevant sections from the manuscript (include quotes in quotation marks "like this" to indicate direct quotes from your manuscript), or elaborate on this item by providing additional information not in the ms, or briefly explain why the item is not applicable/relevant for your study

The intervention design and content was reported on in detail in Janowski et al. (2023) and is summarize in the current paper as follows:

"ParentApp is a smartphone app adapted from the in-person, group-based PLH Teens program, which was originally developed in South Africa..."

"The app, which is open-source and non-commercialized, was specifically designed for offline functionality and is compatible with Android devices running version 5.5.1 or later. The content is delivered through text, images, and audio in 12 sessions which mirror the in-person program in a condensed format"

"Skills are reinforced through interactive activities within sessions as well as skill-building home practice activities aimed at helping caregivers apply new skills with their adolescents"

"A habit-tracking tool is used to log positive parenting and mental health promoting behaviors, and caregivers have access to a library with essential parenting tips, local support resources, and technical support"

Experimental factors are described in the paper as follows:

Factor A: Guidance (Self-guided, Guided)
"In this trial, half of the clusters received no human support post onboarding, while the remaining half received guidance via facilitator-moderated WhatsApp groups. The purpose of these groups was to promote social connection, and sustain caregiver engagement in ParentApp. Each cluster assigned to the guided factor level had one corresponding WhatsApp group, with groups overseen by one lead facilitator and one co-facilitator (totaling 8 groups and 8 facilitators). Facilitators, locally recruited paraprofessionals with prior experience supporting caregivers remotely during the Tanzanian pilot, were trained by Clowns Without Borders South Africa, an NGO with experience leading training of facilitators for PLH programs. A facilitator manual provided instruction on managing the groups and included standardized messages for each module. Facilitators monitored caregiver discussions, shared helpful tips and reminders, addressed questions raised by participants, and led weekly 1-hour live chat sessions to encourage discussion and reflection"

Factor B: App Design (Structured, Unstructured)
"In this trial, two versions of the app design were tested (see screenshot examples in Figure 1 and Figure 2). Half of the clusters were assigned structured, sequential access to the app's content. This design involved releasing a new module every 7 days over a 12-week period. Content-related notifications aligned with the module release schedule, independent of user engagement. Home practice activity reviews, which prompt participants to reflect on their experiences with the module's home practice, automatically unlocked at the end of the 7-day cycle for modules 2 through 11. This structured design retained the generic cartoon illustrations from earlier testing phases. The remaining clusters were allocated unstructured, non-sequential access to content and activities immediately after app installation. This design allowed participants to navigate through the 12 modules at their own pace and in their preferred order. Modules were subdivided into shorter tasks for easier navigation, with home practice activity reviews presented as the final task for modules 2 through 11. Content-related notifications and reminders were disabled in this design, as users could access content without restrictions. The unstructured design replaced the generic cartoon images with culturally adapted illustrations featuring human-like characters tailored to the Tanzanian context"

Factor C: Digital Support (Basic, Enhanced)
"In this trial, all clusters were invited to attend a basic ParentApp-focused digital support training at the in-person onboarding session (also described above). This included an embedded tutorial on app navigation, followed by a group-based orientation of ParentApp, including demonstrations and practice opportunities. Additionally, half of the clusters were invited to attend a 15-20-minute enhanced support training aimed at improving general smartphone literacy and boosting participant confidence in using various smartphone features beyond the app itself. The training curriculum, developed by INNODEMS Kenya, an NGO providing technical support during trial implementation, covered downloading and installing useful apps from Google Play, effective information seeking and internet usage (eg, utilizing simple search terms in a browser and strategies for conserving mobile data), and online safety and awareness. Research assistants provided the enhanced digital training at the end of the in-person onboarding session"

5-ix) Describe use parameters

Describe use parameters (e.g., intended “doses” and optimal timing for use). Clarify what instructions or recommendations were given to the user, e.g., regarding timing, frequency, heaviness of use, if any, or was the intervention used ad libitum.

	1	2	3	4	5	
subitem not at all important	○	○	◉	○	○	essential

Clear selection

Does your paper address subitem 5-ix?

Copy and paste relevant sections from the manuscript (include quotes in quotation marks "like this" to indicate direct quotes from your manuscript), or elaborate on this item by providing additional information not in the ms, or briefly explain why the item is not applicable/relevant for your study

Program use was left up to participants, although they were encouraged to return to the app as often as needed. The structured app was designed for weekly use, while the unstructured app was designed to allow participants to use the app in their own time and at their own pace.

5-x) Clarify the level of human involvement

Clarify the level of human involvement (care providers or health professionals, also technical assistance) in the e-intervention or as co-intervention (detail number and expertise of professionals involved, if any, as well as "type of assistance offered, the timing and frequency of the support, how it is initiated, and the medium by which the assistance is delivered". It may be necessary to distinguish between the level of human involvement required for the trial, and the level of human involvement required for a routine application outside of a RCT setting (discuss under item 21 – generalizability).

	1	2	3	4	5	
subitem not at all important	○	○	○	○	◉	essential

Clear selection

Does your paper address subitem 5-x?

Copy and paste relevant sections from the manuscript (include quotes in quotation marks "like this" to indicate direct quotes from your manuscript), or elaborate on this item by providing additional information not in the ms, or briefly explain why the item is not applicable/relevant for your study

The paper states: "....caregivers participated in an [in-person] app orientation training, which covered the app's key features and the first session"

The paper also states:" Additionally, half of the clusters were invited to attend a 15-20-minute enhanced support training aimed at improving general smartphone literacy". This took place at the end of in-person onboarding.

In addition the the app onboarding, the following, half of the clusters also received facilitator-moderated guidance:

"...half of the clusters received no human support following the in-person orientation and onboarding session. The remaining clusters received guidance from facilitators via WhatsApp groups"

5-xi) Report any prompts/reminders used

Report any prompts/reminders used: Clarify if there were prompts (letters, emails, phone calls, SMS) to use the application, what triggered them, frequency etc. It may be necessary to distinguish between the level of prompts/reminders required for the trial, and the level of prompts/reminders for a routine application outside of a RCT setting (discuss under item 21 – generalizability).

	1	2	3	4	5	
subitem not at all important	○	○	○	○	◉	essential

Clear selection

Does your paper address subitem 5-xi? *

Copy and paste relevant sections from the manuscript (include quotes in quotation marks "like this" to indicate direct quotes from your manuscript), or elaborate on this item by providing additional information not in the ms, or briefly explain why the item is not applicable/relevant for your study

Prompts are described in the study as such: "To encourage continued usage, three types of push notifications are sent to participants: one scheduled within the first day of the user's initial app launch, another scheduled 6 days after the user's last app launch, and a third scheduled 30 days after the user's last app launch."

An SMS was also sent to all study participants at the end of the study to assist data synchronization with the cloud server: "Efforts to address this included sending participants a reminder SMS towards the end of the study, along with an additional data bundle"

5-xii) Describe any co-interventions (incl. training/support)

Describe any co-interventions (incl. training/support): Clearly state any interventions that are provided in addition to the targeted eHealth intervention, as ehealth intervention may not be designed as stand-alone intervention. This includes training sessions and support [1]. It may be necessary to distinguish between the level of training required for the trial, and the level of training for a routine application outside of a RCT setting (discuss under item 21 – generalizability.

	1	2	3	4	5	
subitem not at all important	○	○	○	○	●	essential

Clear selection

Does your paper address subitem 5-xii? *

Copy and paste relevant sections from the manuscript (include quotes in quotation marks "like this" to indicate direct quotes from your manuscript), or elaborate on this item by providing additional information not in the ms, or briefly explain why the item is not applicable/relevant for your study

The paper states: "....caregivers participated in an [in-person] app orientation training, which covered the app's key features and the first session"

Experimental factors are described in the paper as follows:

Factor A: Guidance (Self-guided, Guided)
"In this trial, half of the clusters received no human support post onboarding, while the remaining half received guidance via facilitator-moderated WhatsApp groups. The purpose of these groups was to promote social connection, and sustain caregiver engagement in ParentApp. Each cluster assigned to the guided factor level had one corresponding WhatsApp group, with groups overseen by one lead facilitator and one co-facilitator (totaling 8 groups and 8 facilitators). Facilitators, locally recruited paraprofessionals with prior experience supporting caregivers remotely during the Tanzanian pilot, were trained by Clowns Without Borders South Africa, an NGO with experience leading training of facilitators for PLH programs. A facilitator manual provided instruction on managing the groups and included standardized messages for each module. Facilitators monitored caregiver discussions, shared helpful tips and reminders, addressed questions raised by participants, and led weekly 1-hour live chat sessions to encourage discussion and reflection"

Factor B: App Design (Structured, Unstructured)
"In this trial, two versions of the app design were tested (see screenshot examples in Figure 1 and Figure 2). Half of the clusters were assigned structured, sequential access to the app's content. This design involved releasing a new module every 7 days over a 12-week period. Content-related notifications aligned with the module release schedule, independent of user engagement. Home practice activity reviews, which prompt participants to reflect on their experiences with the module's home practice, automatically unlocked at the end of the 7-day cycle for modules 2 through 11. This structured design retained the generic cartoon illustrations from earlier testing phases. The remaining clusters were allocated unstructured, non-sequential access to content and activities immediately after app installation. This design allowed participants to navigate through the 12 modules at their own pace and in their preferred order. Modules were subdivided into shorter tasks for easier navigation, with home practice activity reviews presented as the final task for modules 2 through 11. Content-related notifications and reminders were disabled in this design, as users could access content without restrictions. The unstructured design replaced the generic cartoon images with culturally adapted illustrations featuring human-like characters tailored to the Tanzanian context"

Factor C: Digital Support (Basic, Enhanced)
"In this trial, all clusters were invited to attend a basic ParentApp-focused digital support training at the in-person onboarding session (also described above). This included an embedded tutorial on app navigation, followed by a group-based orientation of ParentApp, including demonstrations and practice opportunities. Additionally, half of the clusters were invited to attend a 15-20-minute enhanced support training aimed at improving general smartphone literacy and boosting participant confidence in using various smartphone features beyond the app itself. The training curriculum, developed by INNODEMS Kenya, an NGO providing technical support during trial implementation, covered downloading and installing useful apps from Google Play, effective information seeking and internet usage (eg, utilizing simple search terms in a browser and strategies for conserving mobile data), and online safety and awareness. Research assistants provided the enhanced digital training at the end of the in-person onboarding session"

6a) Completely defined pre-specified primary and secondary outcome measures, including how and when they were assessed

Does your paper address CONSORT subitem 6a? *

Copy and paste relevant sections from the manuscript (include quotes in quotation marks "like this" to indicate direct quotes from your manuscript), or elaborate on this item by providing additional information not in the ms, or briefly explain why the item is not applicable/relevant for your study

The primary and secondary outcomes in this paper were program engagement metrics. These are described in the Measure and Assessment section as such:

"Primary Outcome: Three primary engagement outcomes were evaluated based on the three-phase definition outlined above: (1) the number of app launches throughout the intervention period, (2) the number of completed modules out of 12, and (3) the number of home practice activities reviewed out of 10."

"Secondary Outcomes: Three secondary engagement outcomes were assessed: (1) the amount of time spent on the app during the intervention period, (2) the number of modules started out of 12, and (3) the number of self-reported positive parenting/mental health promoting habits logged throughout the intervention."

6a-i) Online questionnaires: describe if they were validated for online use and apply CHERRIES items to describe how the questionnaires were designed/deployed

If outcomes were obtained through online questionnaires, describe if they were validated for online use and apply CHERRIES items to describe how the questionnaires were designed/deployed [9].

	1	2	3	4	5	
subitem not at all important	○	○	○	○	◉	essential

Clear selection

Does your paper address subitem 6a-i?

Copy and paste relevant sections from manuscript text

No outcomes in this paper were collected using online questionnaires. Baseline demographic and behavioural characteristics were collected via a self-administered embedded questionnaire. The paper states the following:

"Self-report demographic and behavioral data were collected at baseline using a self-administered embedded questionnaire. All measures underwent translation into Kiswahili, followed by back-translation, and were previously used in Tanzania [54]. Behavioral measures were non-proprietary, freely available, and recommended by the GPI."

6a-ii) Describe whether and how “use” (including intensity of use/dosage) was defined/measured/monitored

Describe whether and how “use” (including intensity of use/dosage) was defined/measured/monitored (logins, logfile analysis, etc.). Use/adoption metrics are important process outcomes that should be reported in any ehealth trial.

	1	2	3	4	5	
subitem not at all important	○	○	○	○	◉	essential

Clear selection

Does your paper address subitem 6a-ii?

Copy and paste relevant sections from manuscript text

As included in the paper, "Engagement was operationalized as a three-phase process: (1) engagement with the app, (2) engagement with the content, and (3) engagement with the activities. This definition aligns with established literature and emphasizes the multi-phase nature of engaging with digital interventions [28,29,56]. Automated tracking was used to collect engagement data each time a participant interacted with the app. Tracking records, including module and activity completion rates, type and frequency of content viewed, and responses to in-app tasks and the baseline assessment, were seamlessly uploaded to a Metabase cloud server whenever participants’ devices accessed the internet. Additional usage data was automatically tracked through Matomo Analytics, capturing date and time stamps of each visit, duration of time spent during each visit, and the frequency of actions or pages viewed."

6a-iii) Describe whether, how, and when qualitative feedback from participants was obtained

Describe whether, how, and when qualitative feedback from participants was obtained (e.g., through emails, feedback forms, interviews, focus groups).

	1	2	3	4	5	
subitem not at all important	○	○	○	○	◉	essential

Clear selection

Does your paper address subitem 6a-iii?

Copy and paste relevant sections from manuscript text

No applicable. Although qualitative data was collected as part of the study, there is no qualitative feedback relevant to this paper.

6b) Any changes to trial outcomes after the trial commenced, with reasons

Does your paper address CONSORT subitem 6b? *

Copy and paste relevant sections from the manuscript (include quotes in quotation marks "like this" to indicate direct quotes from your manuscript), or elaborate on this item by providing additional information not in the ms, or briefly explain why the item is not applicable/relevant for your study

One modification was made to one of the outcomes. The paper states the following: "Primary outcome 3 underwent a post-hoc modification from the protocol. Originally intended to measure user access to the home practice tab for activity instructions, it now evaluates whether users started the post-activity review process. This adjustment aims to strengthen the outcome by providing a more comprehensive measure of participant engagement with the activity."

7a) How sample size was determined

NPT: When applicable, details of whether and how the clustering by care provides or centers was addressed

7a-i) Describe whether and how expected attrition was taken into account when calculating the sample size

Describe whether and how expected attrition was taken into account when calculating the sample size.

	1	2	3	4	5	
subitem not at all important	○	○	○	○	◉	essential

Clear selection

Does your paper address subitem 7a-i?

Copy and paste relevant sections from manuscript title (include quotes in quotation marks "like this" to indicate direct quotes from your manuscript), or elaborate on this item by providing additional information not in the ms, or briefly explain why the item is not applicable/relevant for your study

The sample size considerations for this study have previously been described in detail in our published protocol (Janowski et al., 2023). They are also summarised in the current paper as follows: "An a priori simulation-based power analysis determined that to ensure adequate statistical power, 640 caregivers across 16 clusters were needed, based on an average cluster size of 40 [49]. This cluster size was chosen with future scalability in mind, as larger WhatsApp groups offer greater reach and cost-effectiveness. Power was set at 80% while taking into account clustering at the sub-ward level, the potential impact of participant-level covariates, and an alpha level of 0.05."

7b) When applicable, explanation of any interim analyses and stopping guidelines

Does your paper address CONSORT subitem 7b? *

Copy and paste relevant sections from the manuscript (include quotes in quotation marks "like this" to indicate direct quotes from your manuscript), or elaborate on this item by providing additional information not in the ms, or briefly explain why the item is not applicable/relevant for your study

We did not conduct interim analyses and there were no stopping guidelines to report.

8a) Method used to generate the random allocation sequence
NPT: When applicable, how care providers were allocated to each trial group

Does your paper address CONSORT subitem 8a? *

Copy and paste relevant sections from the manuscript (include quotes in quotation marks "like this" to indicate direct quotes from your manuscript), or elaborate on this item by providing additional information not in the ms, or briefly explain why the item is not applicable/relevant for your study

Allocation to group assignment is described in the paper as follows: "A list of eligible clusters was then sent to an off-site research team member for randomization. Randomization involved a single-stage randomization process, assigning each cluster to one of the 8 combinations of experimental conditions using a 1:1 allocation ratio. The random sequence, generated using a randomization algorithm in R, was then sent back to the in-country research team."

8b) Type of randomisation; details of any restriction (such as blocking and block size)

Does your paper address CONSORT subitem 8b? *

Copy and paste relevant sections from the manuscript (include quotes in quotation marks "like this" to indicate direct quotes from your manuscript), or elaborate on this item by providing additional information not in the ms, or briefly explain why the item is not applicable/relevant for your study

The type of randomisation is described as follows in the paper: "Randomization involved a single-stage randomization process, assigning each cluster to one of the 8 combinations of experimental conditions using a 1:1 allocation ratio."

9) Mechanism used to implement the random allocation sequence (such as sequentially numbered containers), describing any steps taken to conceal the sequence until interventions were assigned

Does your paper address CONSORT subitem 9? *

Copy and paste relevant sections from the manuscript (include quotes in quotation marks "like this" to indicate direct quotes from your manuscript), or elaborate on this item by providing additional information not in the ms, or briefly explain why the item is not applicable/relevant for your study

As described in the paper, we used an R generated randomisation algorithm: "The random sequence, generated using a randomization algorithm in R..."

10) Who generated the random allocation sequence, who enrolled participants, and who assigned participants to interventions

Does your paper address CONSORT subitem 10? *

Copy and paste relevant sections from the manuscript (include quotes in quotation marks "like this" to indicate direct quotes from your manuscript), or elaborate on this item by providing additional information not in the ms, or briefly explain why the item is not applicable/relevant for your study

As described in the paper, random allocation was conducted by an off-site research team member who had no knowledge of the clusters being randomised, "the list was sent to an off-site research team member for randomization..."

Participants were enrolled by trained research assistants, "At the in-person session, caregivers received information about the program, and research assistants conducted eligibility screening and obtained written informed consent."

Clusters rather than individual participants were assigned to the 8 combinations of experimental conditions based on cluster randomisation.

11a) If done, who was blinded after assignment to interventions (for example, participants, care providers, those assessing outcomes) and how

NPT: Whether or not administering co-interventions were blinded to group assignment

11a-i) Specify who was blinded, and who wasn't

Specify who was blinded, and who wasn't. Usually, in web-based trials it is not possible to blind the participants [1, 3] (this should be clearly acknowledged), but it may be possible to blind outcome assessors, those doing data analysis or those administering co-interventions (if any).

	1	2	3	4	5	
subitem not at all important	○	○	○	○	◉	essential

Clear selection

Does your paper address subitem 11a-i? *

Copy and paste relevant sections from the manuscript (include quotes in quotation marks "like this" to indicate direct quotes from your manuscript), or elaborate on this item by providing additional information not in the ms, or briefly explain why the item is not applicable/relevant for your study

Participants were blinded to group assignment. The paper states, "During the onboarding process, participants were only informed about the experimental conditions to which their cluster was assigned."

11a-ii) Discuss e.g., whether participants knew which intervention was the "intervention of interest" and which one was the "comparator"

Informed consent procedures (4a-ii) can create biases and certain expectations - discuss e.g., whether participants knew which intervention was the "intervention of interest" and which one was the "comparator".

	1	2	3	4	5	
subitem not at all important	○	○	○	○	◉	essential

Clear selection

Does your paper address subitem 11a-ii?

Copy and paste relevant sections from the manuscript (include quotes in quotation marks "like this" to indicate direct quotes from your manuscript), or elaborate on this item by providing additional information not in the ms, or briefly explain why the item is not applicable/relevant for your study

This is not relevant in the current study. All participants were randomized to one of 8 different combinations of each of the 3 experimental factors of interest. Within each factor, one half of the clusters were randomized to each factor level, allowing each factor level to be compared to one another. This full factorial design means that no participants received a traditional "control" intervention. All received various combinations of the experimental factors of interest.

11b) If relevant, description of the similarity of interventions

(this item is usually not relevant for ehealth trials as it refers to similarity of a placebo or sham intervention to a active medication/intervention)

Does your paper address CONSORT subitem 11b? *

Copy and paste relevant sections from the manuscript (include quotes in quotation marks "like this" to indicate direct quotes from your manuscript), or elaborate on this item by providing additional information not in the ms, or briefly explain why the item is not applicable/relevant for your study

Not relevant for this study.

12a) Statistical methods used to compare groups for primary and secondary outcomes

NPT: When applicable, details of whether and how the clustering by care providers or centers was addressed

Does your paper address CONSORT subitem 12a? *

Copy and paste relevant sections from the manuscript (include quotes in quotation marks "like this" to indicate direct quotes from your manuscript), or elaborate on this item by providing additional information not in the ms, or briefly explain why the item is not applicable/relevant for your study

The statistical methods are described as follows: "Analyses used Generalized Linear Mixed Effects Models (GLMMs) with a Poisson distribution to estimate the impact of experimental factors on engagement. Models included a random intercept term to account for nesting of participants within clusters. Analyses were intention-to-treat, but only included participants who successfully installed ParentApp, as data collection was contingent upon app installation."

12a-i) Imputation techniques to deal with attrition / missing values

Imputation techniques to deal with attrition / missing values: Not all participants will use the intervention/comparator as intended and attrition is typically high in ehealth trials. Specify how participants who did not use the application or dropped out from the trial were treated in the statistical analysis (a complete case analysis is strongly discouraged, and simple imputation techniques such as LOCF may also be problematic [4]).

	1	2	3	4	5	
subitem not at all important	○	○	○	○	●	essential

Clear selection

Does your paper address subitem 12a-i? *

Copy and paste relevant sections from the manuscript (include quotes in quotation marks "like this" to indicate direct quotes from your manuscript), or elaborate on this item by providing additional information not in the ms, or briefly explain why the item is not applicable/relevant for your study

Imputation of missing baseline data is described as follows: "Missingness in caregiver baseline data (5% to 7.5% at item level) was addressed using multiple imputation with fully conditional specification [63]. A total of 10 imputations were conducted using predictive mean matching. Clustering was accounted for by specifying -2 in the predictor matrix. Imputation at the item level was passive, summing behavioral items to generate total scores within the imputation process."

12b) Methods for additional analyses, such as subgroup analyses and adjusted analyses

Does your paper address CONSORT subitem 12b? *

Copy and paste relevant sections from the manuscript (include quotes in quotation marks "like this" to indicate direct quotes from your manuscript), or elaborate on this item by providing additional information not in the ms, or briefly explain why the item is not applicable/relevant for your study

Adjusted analyses are described as follows: "Models were adjusted for the prespecified covariates: caregiver age, caregiver gender, financial stress, food insecurity, child maltreatment (physical and emotional abuse), positive parenting involvement and supervision, parental depression, and parenting stress."

X26) REB/IRB Approval and Ethical Considerations [recommended as subheading under "Methods"] (not a CONSORT item)

X26-i) Comment on ethics committee approval

	1	2	3	4	5	
subitem not at all important	○	○	○	○	◉	essential

Clear selection

Does your paper address subitem X26-i?

Copy and paste relevant sections from the manuscript (include quotes in quotation marks "like this" to indicate direct quotes from your manuscript), or elaborate on this item by providing additional information not in the ms, or briefly explain why the item is not applicable/relevant for your study

Yes, the paper includes this under the heading Ethics and Informed Consent: "Ethical approval was granted by the Departmental Research Ethics Committee at the University of Oxford (Ref: R69744/RE001) and the Tanzanian National Institute of Medical Research (Ref: NIMR/HQ/R.8s/Vol.IX/3856)."

x26-ii) Outline informed consent procedures

Outline informed consent procedures e.g., if consent was obtained offline or online (how? Checkbox, etc.?), and what information was provided (see 4a-ii). See [6] for some items to be included in informed consent documents.

	1	2	3	4	5	
subitem not at all important	○	○	○	○	●	essential

Clear selection

Does your paper address subitem X26-ii?

Copy and paste relevant sections from the manuscript (include quotes in quotation marks "like this" to indicate direct quotes from your manuscript), or elaborate on this item by providing additional information not in the ms, or briefly explain why the item is not applicable/relevant for your study

Informed consent is described as follows: "Written informed consent was obtained from all participants at the in-person orientation and onboarding session. Participants were also required to accept the app's Terms and Conditions and Privacy Policy, which contained information about the study and how personal information from the app will be collected, used, and shared"

X26-iii) Safety and security procedures

Safety and security procedures, incl. privacy considerations, and any steps taken to reduce the likelihood or detection of harm (e.g., education and training, availability of a hotline)

	1	2	3	4	5	
subitem not at all important	○	○	○	●	○	essential

Clear selection

Does your paper address subitem X26-iii?

Copy and paste relevant sections from the manuscript (include quotes in quotation marks "like this" to indicate direct quotes from your manuscript), or elaborate on this item by providing additional information not in the ms, or briefly explain why the item is not applicable/relevant for your study

Even though this is a low-risk intervention (there were no safety concerns in the ParentApp pilot study or any of the in-person Parenting for Lifelong Health effectiveness trials), local support resources for self-referral were provided within the app.

The published protocol provides a detailed description: "the app includes local self-referral and emergency contacts which are affordable and remote-friendly. This information will include services for family and child support, substance use, gender-based violence and rape, child abuse and protection, physical, mental, and contact details for available helplines. These resources were identified by the research team following a thorough mapping of online and affordable support systems in Tanzania."

RESULTS

13a) For each group, the numbers of participants who were randomly assigned, received intended treatment, and were analysed for the primary outcome

NPT: The number of care providers or centers performing the intervention in each group and the number of patients treated by each care provider in each center

Does your paper address CONSORT subitem 13a? *

Copy and paste relevant sections from the manuscript (include quotes in quotation marks "like this" to indicate direct quotes from your manuscript), or elaborate on this item by providing additional information not in the ms, or briefly explain why the item is not applicable/relevant for your study

The paper clearly outlines that half of the participants were randomized to each experimental factor level. Figure 3 depicts a CONSORT diagram,
indicating the number of randomized clusters, the number of participants exposed to the intervention, and the number who were analyzed.

13b) For each group, losses and exclusions after randomisation, together with reasons

Does your paper address CONSORT subitem 13b? (NOTE: Preferably, this is shown in a CONSORT flow diagram) *

Copy and paste relevant sections from the manuscript (include quotes in quotation marks "like this" to indicate direct quotes from your manuscript), or elaborate on this item by providing additional information not in the ms, or briefly explain why the item is not applicable/relevant for your study

As depicted in the CONSORT diagram and discussed in the analyses, all participants installed the app included in the analyses.

13b-i) Attrition diagram

Strongly recommended: An attrition diagram (e.g., proportion of participants still logging in or using the intervention/comparator in each group plotted over time, similar to a survival curve) or other figures or tables demonstrating usage/dose/engagement.

	1	2	3	4	5	
subitem not at all important	○	○	◉	○	○	essential

Clear selection

Does your paper address subitem 13b-i?

Copy and paste relevant sections from the manuscript or cite the figure number if applicable (include quotes in quotation marks "like this" to indicate direct quotes from your manuscript), or elaborate on this item by providing additional information not in the ms, or briefly explain why the item is not applicable/relevant for your study

We do not include an attrition diagram because it is not relevant to the current analyses. We explored program engagement among all participants who installed the app using automated user data on app use. Participants who failed to use the app were included in the analyses, so there is no attrition in the main outcomes to be accounted for.

14a) Dates defining the periods of recruitment and follow-up

Does your paper address CONSORT subitem 14a? *

Copy and paste relevant sections from the manuscript (include quotes in quotation marks "like this" to indicate direct quotes from your manuscript), or elaborate on this item by providing additional information not in the ms, or briefly explain why the item is not applicable/relevant for your study

As reported in the paper, "Participant recruitment and onboarding activities began on October 24, 2022, and concluded on December 1, 2022".

"The final dataset was extracted from Metabase and Matomo Analytics in March 2023, approximately four months after the last cluster of participants enrolled in the study."

14a-i) Indicate if critical “secular events” fell into the study period

Indicate if critical “secular events” fell into the study period, e.g., significant changes in Internet resources available or “changes in computer hardware or Internet delivery resources”

	1	2	3	4	5	
subitem not at all important	○	○	○	◉	○	essential

Clear selection

Does your paper address subitem 14a-i?

Copy and paste relevant sections from the manuscript (include quotes in quotation marks "like this" to indicate direct quotes from your manuscript), or elaborate on this item by providing additional information not in the ms, or briefly explain why the item is not applicable/relevant for your study

There were no known critical "secular events" during the study period to report.

14b) Why the trial ended or was stopped (early)

Does your paper address CONSORT subitem 14b? *

Copy and paste relevant sections from the manuscript (include quotes in quotation marks "like this" to indicate direct quotes from your manuscript), or elaborate on this item by providing additional information not in the ms, or briefly explain why the item is not applicable/relevant for your study

Not relevant - the study was not ended early.

15) A table showing baseline demographic and clinical characteristics for each group

NPT: When applicable, a description of care providers (case volume, qualification, expertise, etc.) and centers (volume) in each group

Does your paper address CONSORT subitem 15? *

Copy and paste relevant sections from the manuscript (include quotes in quotation marks "like this" to indicate direct quotes from your manuscript), or elaborate on this item by providing additional information not in the ms, or briefly explain why the item is not applicable/relevant for your study

This is included in Table 3.

15-i) Report demographics associated with digital divide issues

In ehealth trials it is particularly important to report demographics associated with digital divide issues, such as age, education, gender, social-economic status, computer/Internet/ehealth literacy of the participants, if known.

	1	2	3	4	5	
subitem not at all important	○	○	○	◉	○	essential

Clear selection

Does your paper address subitem 15-i? *

Copy and paste relevant sections from the manuscript (include quotes in quotation marks "like this" to indicate direct quotes from your manuscript), or elaborate on this item by providing additional information not in the ms, or briefly explain why the item is not applicable/relevant for your study

Table 3 includes age, gender, household composition, food insecurity, and adolescent orphanhood status. As outlined above, as a condition of enrollment, all participants came from socioeconomically vulnerable communities but had to have regular access to a smartphone.

16) For each group, number of participants (denominator) included in each analysis and whether the analysis was by original assigned groups

16-i) Report multiple “denominators” and provide definitions

Report multiple “denominators” and provide definitions: Report N’s (and effect sizes) “across a range of study participation [and use] thresholds” [1], e.g., N exposed, N consented, N used more than x times, N used more than y weeks, N participants “used” the intervention/comparator at specific pre-defined time points of interest (in absolute and relative numbers per group). Always clearly define “use” of the intervention.

	1	2	3	4	5	
subitem not at all important	○	○	○	○	◉	essential

Clear selection

Does your paper address subitem 16-i? *

Copy and paste relevant sections from the manuscript (include quotes in quotation marks "like this" to indicate direct quotes from your manuscript), or elaborate on this item by providing additional information not in the ms, or briefly explain why the item is not applicable/relevant for your study

As reflected in Figure 3 and discussed in the the results, all participants who installed the app, regardless of app usage, were included in analyses. Supplementary analyses found no significant effect of cluster randomization to experimental conditions on app installation status.

16-ii) Primary analysis should be intent-to-treat

Primary analysis should be intent-to-treat, secondary analyses could include comparing only “users”, with the appropriate caveats that this is no longer a randomized sample (see 18-i).

	1	2	3	4	5	
subitem not at all important	○	○	○	○	◉	essential

Clear selection

Does your paper address subitem 16-ii?

Copy and paste relevant sections from the manuscript (include quotes in quotation marks "like this" to indicate direct quotes from your manuscript), or elaborate on this item by providing additional information not in the ms, or briefly explain why the item is not applicable/relevant for your study

As stated in the paper, "A total of 614 participants installed the app, all of whom were included in intention-to-treat analyses."

17a) For each primary and secondary outcome, results for each group, and the estimated effect size and its precision (such as 95% confidence interval)

Does your paper address CONSORT subitem 17a? *

Copy and paste relevant sections from the manuscript (include quotes in quotation marks "like this" to indicate direct quotes from your manuscript), or elaborate on this item by providing additional information not in the ms, or briefly explain why the item is not applicable/relevant for your study

Effect sizes and confidence intervals are reported for each primary and secondary outcome, both in text and in Table 5 and Table 6.

17a-i) Presentation of process outcomes such as metrics of use and intensity of use

In addition to primary/secondary (clinical) outcomes, the presentation of process outcomes such as metrics of use and intensity of use (dose, exposure) and their operational definitions is critical. This does not only refer to metrics of attrition (13-b) (often a binary variable), but also to more continuous exposure metrics such as “average session length”. These must be accompanied by a technical description how a metric like a “session” is defined (e.g., timeout after idle time) [1] (report under item 6a).

	1	2	3	4	5	
subitem not at all important	○	○	○	○	◉	essential

Clear selection

Does your paper address subitem 17a-i?

Copy and paste relevant sections from the manuscript (include quotes in quotation marks "like this" to indicate direct quotes from your manuscript), or elaborate on this item by providing additional information not in the ms, or briefly explain why the item is not applicable/relevant for your study

The main outcomes in this paper were the metrics of program use/engagement, as described above. Thus, we report in detail on program engagement for the entire sample and by factor level, but these are presented as main outcomes, not process measures.

17b) For binary outcomes, presentation of both absolute and relative effect sizes is recommended

Does your paper address CONSORT subitem 17b? *

Copy and paste relevant sections from the manuscript (include quotes in quotation marks "like this" to indicate direct quotes from your manuscript), or elaborate on this item by providing additional information not in the ms, or briefly explain why the item is not applicable/relevant for your study

Raw means and their standard deviations are provided for all outcomes in Table 4. Effect sizes along with their corresponding 95% confidence intervals were calculated as mean ratios (MR), where MR = exp(2 * β), with β representing the unstandardized regression coefficient. Unstandardized regression coefficients derived from effect-coded experimental factors.

18) Results of any other analyses performed, including subgroup analyses and adjusted analyses, distinguishing pre-specified from exploratory

Does your paper address CONSORT subitem 18? *

Copy and paste relevant sections from the manuscript (include quotes in quotation marks "like this" to indicate direct quotes from your manuscript), or elaborate on this item by providing additional information not in the ms, or briefly explain why the item is not applicable/relevant for your study

We clearly present the interaction effect analyses as exploratory "In exploratory analyses, all three factors were included as main effects alongside their two-way interactions, while adjusting for baseline characteristics".

18-i) Subgroup analysis of comparing only users

A subgroup analysis of comparing only users is not uncommon in ehealth trials, but if done, it must be stressed that this is a self-selected sample and no longer an unbiased sample from a randomized trial (see 16-iii).

	1	2	3	4	5	
subitem not at all important	○	○	○	◉	○	essential

Clear selection

Does your paper address subitem 18-i?

Copy and paste relevant sections from the manuscript (include quotes in quotation marks "like this" to indicate direct quotes from your manuscript), or elaborate on this item by providing additional information not in the ms, or briefly explain why the item is not applicable/relevant for your study

Not relevant to the current paper.

19) All important harms or unintended effects in each group
(for specific guidance see CONSORT for harms)

Does your paper address CONSORT subitem 19? *

Copy and paste relevant sections from the manuscript (include quotes in quotation marks "like this" to indicate direct quotes from your manuscript), or elaborate on this item by providing additional information not in the ms, or briefly explain why the item is not applicable/relevant for your study

There were no harms or unintended effects to report. Two interaction effects resulted in a slight reduction of app use and clearly stated in the text. However, this was not deemed a harm by the researchers, especially because the reduction was slight and only observed for one out of the six engagement outcomes.

19-i) Include privacy breaches, technical problems

Include privacy breaches, technical problems. This does not only include physical “harm” to participants, but also incidents such as perceived or real privacy breaches [1], technical problems, and other unexpected/unintended incidents. “Unintended effects” also includes unintended positive effects [2].

	1	2	3	4	5	
subitem not at all important	○	○	○	○	◉	essential

Clear selection

Does your paper address subitem 19-i?

Copy and paste relevant sections from the manuscript (include quotes in quotation marks "like this" to indicate direct quotes from your manuscript), or elaborate on this item by providing additional information not in the ms, or briefly explain why the item is not applicable/relevant for your study

There were no privacy breaches to report. Technical problems including internet instability and phone-to-app incompatibility are discussed in the text and in presented in Figure 3.

19-ii) Include qualitative feedback from participants or observations from staff/researchers

Include qualitative feedback from participants or observations from staff/researchers, if available, on strengths and shortcomings of the application, especially if they point to unintended/unexpected effects or uses. This includes (if available) reasons for why people did or did not use the application as intended by the developers.

	1	2	3	4	5	
subitem not at all important	○	○	○	○	◉	essential

Clear selection

Does your paper address subitem 19-ii?

Copy and paste relevant sections from the manuscript (include quotes in quotation marks "like this" to indicate direct quotes from your manuscript), or elaborate on this item by providing additional information not in the ms, or briefly explain why the item is not applicable/relevant for your study

There is no qualitative feedback relevant to this paper to report.

DISCUSSION

22) Interpretation consistent with results, balancing benefits and harms, and considering other relevant evidence

NPT: In addition, take into account the choice of the comparator, lack of or partial blinding, and unequal expertise of care providers or centers in each group

22-i) Restate study questions and summarize the answers suggested by the data, starting with primary outcomes and process outcomes (use)

Restate study questions and summarize the answers suggested by the data, starting with primary outcomes and process outcomes (use).

	1	2	3	4	5	
subitem not at all important	○	○	○	○	◉	essential

Clear selection

Does your paper address subitem 22-i? *

Copy and paste relevant sections from the manuscript (include quotes in quotation marks "like this" to indicate direct quotes from your manuscript), or elaborate on this item by providing additional information not in the ms, or briefly explain why the item is not applicable/relevant for your study

The discussion states: "This is the first known factorial trial of a digital parenting intervention for families with adolescents in a LMIC. It follows MOST principles [47] to optimize engagement with an app targeting socioeconomically vulnerable families in Tanzania. Findings offer important insights into engagement-enhancing intervention design and implementation strategies for digital parenting interventions tailored to the unique contexts and needs of LMIC settings."

Under Principal Findings, the paper discusses the main effects of the factors on primary and secondary outcomes, along with relevant literature and theories.

22-ii) Highlight unanswered new questions, suggest future research

Highlight unanswered new questions, suggest future research.

	1	2	3	4	5	
subitem not at all important	○	○	○	○	◉	essential

Clear selection

Does your paper address subitem 22-ii?

Copy and paste relevant sections from the manuscript (include quotes in quotation marks "like this" to indicate direct quotes from your manuscript), or elaborate on this item by providing additional information not in the ms, or briefly explain why the item is not applicable/relevant for your study

The discussion highlights that "Continued exploration and refinement of these approaches will be instrumental in developing evidence-based, scalable digital interventions capable of effectively addressing parenting challenges and fostering positive outcomes for caregivers and their children in resource-constrained contexts."

20) Trial limitations, addressing sources of potential bias, imprecision, and, if relevant, multiplicity of analyses

20-i) Typical limitations in ehealth trials

Typical limitations in ehealth trials: Participants in ehealth trials are rarely blinded. Ehealth trials often look at a multiplicity of outcomes, increasing risk for a Type I error. Discuss biases due to non-use of the intervention/usability issues, biases through informed consent procedures, unexpected events.

	1	2	3	4	5	
subitem not at all important	○	○	○	○	◉	essential

Clear selection

Does your paper address subitem 20-i? *

Copy and paste relevant sections from the manuscript (include quotes in quotation marks "like this" to indicate direct quotes from your manuscript), or elaborate on this item by providing additional information not in the ms, or briefly explain why the item is not applicable/relevant for your study

There are several trial limitations are considered in this paper. They are discussed in the paper as follows: "Several limitations should be considered when interpreting the study's results. First, this study did not measure the impact of the factors on caregiver or adolescent outcomes. Consequently, the relationship between engagement and improvements in participant outcomes remains uncertain. Additionally, this trial does not permit the evaluation of the app's direct effectiveness. However, functioning as the MOST framework's Optimization Phase within the broader ParentApp study, this trial lays the groundwork for a comprehensive evaluation of these impacts in the forthcoming randomized controlled trial (RCT) [74]. Second, while the study's focus on broader implementation strategies provides valuable insights for implementers and researchers, the generalizability of results to different LMIC settings or populations remains unclear. Further investigation into factors influencing uptake and engagement across diverse behavioral domains, digital platforms, and delivery contexts is needed to enhance the applicability of these strategies in different settings.

Third, data collection via automated tracking required internet access for synchronization with the cloud servers. As a result, participant usage data for those whose 1GB monthly internet bundles were depleted before data synchronization occurred may not have been uploaded, potentially leading to an underestimation of engagement rates. Efforts to address this included sending participants a reminder SMS towards the end of the study, along with an additional data bundle. However, it remains unclear to what extent these measures resolved the issue. Fourth, despite enrolling 680 caregivers, surpassing the desired sample size of 640, technical challenges hindered the installation of the app for a portion of participants (10.3%), with an additional portion (2.1%) failing to access any of the core intervention content even after installation. These challenges were primarily attributed to internet issues and phone-to-app compatibility. Forthcoming qualitative research conducted in parallel to this study highlighted additional barriers to uptake and retention, including the selling of phones due to financial needs, deleting the app due to storage constraints, and program dropout due to severely damaged screens. Despite these challenges, overall content completion rates for participants who started at least one module was 35.57% of the program. While only 7.8% of participants completed all 12 modules, completion rates are consistent with other digital parenting intervention studies in HICs, such as the 7.5% completion rate found in a large-scale study of the online ParentWorks program in Australia [32]. Nonetheless, investigating patterns of early dropout and identifying opportunities for keeping caregivers engaged throughout program delivery remain crucial areas for investigation."

21) Generalisability (external validity, applicability) of the trial findings

NPT: External validity of the trial findings according to the intervention, comparators, patients, and care providers or centers involved in the trial

21-i) Generalizability to other populations

Generalizability to other populations: In particular, discuss generalizability to a general Internet population, outside of a RCT setting, and general patient population, including applicability of the study results for other organizations

	1	2	3	4	5	
subitem not at all important	○	○	○	○	◉	essential

Clear selection

Does your paper address subitem 21-i?

Copy and paste relevant sections from the manuscript (include quotes in quotation marks "like this" to indicate direct quotes from your manuscript), or elaborate on this item by providing additional information not in the ms, or briefly explain why the item is not applicable/relevant for your study

The paper states: "...while the study's focus on broader implementation strategies provides valuable insights for implementers and researchers, the generalizability of results to different LMIC settings or populations remains unclear. Further investigation into factors influencing uptake and engagement across diverse behavioral domains, digital platforms, and delivery contexts is needed to enhance the applicability of these strategies in different settings."

21-ii) Discuss if there were elements in the RCT that would be different in a routine application setting

Discuss if there were elements in the RCT that would be different in a routine application setting (e.g., prompts/reminders, more human involvement, training sessions or other co-interventions) and what impact the omission of these elements could have on use, adoption, or outcomes if the intervention is applied outside of a RCT setting.

	1	2	3	4	5	
subitem not at all important	○	○	○	○	◉	essential

Clear selection

Does your paper address subitem 21-ii?

Copy and paste relevant sections from the manuscript (include quotes in quotation marks "like this" to indicate direct quotes from your manuscript), or elaborate on this item by providing additional information not in the ms, or briefly explain why the item is not applicable/relevant for your study

The paper discusses the strengths of the implementation strategy in relation to future scalability: "Another key strength is the context in which the trial was implemented. The recruitment process drew from a large community sample of families, facilitated by partnering with local gatekeepers and community leaders. This approach closely aligns with how the intervention could be disseminated in the future and adds to the potential sustainability of the program. Additionally, approximately 33% of the caregivers reached in this study were men, a demographic often underrepresented in parenting interventions [67]. Engaging such high levels of men not only extends the generalizability of the results, but represents significant strides towards understanding how hard-to-reach demographics such as men engage in digital parenting interventions. A forthcoming analysis on the predictors of male engagement will provide further insights into this aspect of engagement. Finally, unlike many digital intervention trials that provide participants with digital devices throughout the study, our study's sample consisted of participants who used their own smartphones, including older and low-cost models. This approach not only demonstrates the feasibility of implementing digital interventions in real-world, low-income settings but also suggests potential for cost-effective wide-scale delivery. Furthermore, by deliberately including individuals with varying levels of technological access and proficiency, this study establishes a model for inclusive digital intervention design, particularly in regions like Tanzania where smartphone access is rapidly expanding but still limited [78]."

OTHER INFORMATION

23) Registration number and name of trial registry

Does your paper address CONSORT subitem 23? *

Copy and paste relevant sections from the manuscript (include quotes in quotation marks "like this" to indicate direct quotes from your manuscript), or elaborate on this item by providing additional information not in the ms, or briefly explain why the item is not applicable/relevant for your study

The trial is registered, as cited in the Abstract and Methods. "The trial was preregistered on the Pan-African Clinical Trial Registry (PACTR202210657553944)"

24) Where the full trial protocol can be accessed, if available

Does your paper address CONSORT subitem 24? *

Cite a Multimedia Appendix, other reference, or copy and paste relevant sections from the manuscript (include quotes in quotation marks "like this" to indicate direct quotes from your manuscript), or elaborate on this item by providing additional information not in the ms, or briefly explain why the item is not applicable/relevant for your study

The citation for the trial protocol is: Janowski R, Green O, Shenderovich Y, et al. Optimising engagement in a digital parenting intervention to prevent violence against adolescents in Tanzania: Protocol for a cluster randomised factorial trial. BMC Public Health. 2023;23(1):1224. doi:10.1186/s12889-023-15989-x

25) Sources of funding and other support (such as supply of drugs), role of funders

Does your paper address CONSORT subitem 25? *

Copy and paste relevant sections from the manuscript (include quotes in quotation marks "like this" to indicate direct quotes from your manuscript), or elaborate on this item by providing additional information not in the ms, or briefly explain why the item is not applicable/relevant for your study

The funding statement states: "This study is part of the Global Parenting Initiative, which is funded by the LEGO Foundation, Oak Foundation [OFIL-21-212], the World Childhood Foundation, and The Human Safety Net, the UK Research and Innovation (UKRI) Global Challenges Research Fund Accelerating Achievement for Africa's Adolescents (Accelerate) Hub [ES/S008101/1], the Wellspring Philanthropic Foundation [16204], the European Research Council under the Proof of Concept 2022-1 Call [proposal no. 101067451], and a private family trust which wishes to remain anonymous. RJ was supported by the UKRI Economic and Social Research Council (ESRC) Grand Union Doctoral Training Partnership. YS was supported by DECIPHer and the Wolfson Centre for Young People's Mental Health. DECIPHer is funded by Welsh Government through Health and Care Research Wales. The Wolfson Centre for Young People's Mental Health has been established with support from the Wolfson Foundation."

X27) Conflicts of Interest (not a CONSORT item)

X27-i) State the relation of the study team towards the system being evaluated

In addition to the usual declaration of interests (financial or otherwise), also state the relation of the study team towards the system being evaluated, i.e., state if the authors/evaluators are distinct from or identical with the developers/sponsors of the intervention.

	1	2	3	4	5	
subitem not at all important	○	○	○	○	◉	essential

Clear selection

Does your paper address subitem X27-i?

Copy and paste relevant sections from the manuscript (include quotes in quotation marks "like this" to indicate direct quotes from your manuscript), or elaborate on this item by providing additional information not in the ms, or briefly explain why the item is not applicable/relevant for your study

Conflicts of interest are disclosed as follows: "ParentApp was developed and implemented by members of the research team. RJ’s doctoral research is based on this trial. JML is the CEO and LDC is a voluntary trustee of PLH, a charity based in the United Kingdom and South Africa. JML receives occasional fees for providing training and supervision for PLH programs. Members of the research team have worked on other studies of PLH which contributed funding to their respective universities but not directly to them. The intellectual property of the intervention is owned by the developers of the original intervention under a Creative Commons Attribution-NoDerivatives and NonCommercial 4.0 International Public License. No profit or financial gain will be made from implementation and dissemination of the intervention."

About the CONSORT EHEALTH checklist

As a result of using this checklist, did you make changes in your manuscript? *

- ○ yes, major changes
- ◉ yes, minor changes
- ○ no

What were the most important changes you made as a result of using this checklist?

Including more detailed information on the intervention content and development, and adding screenshot examples of the intervention.

How much time did you spend on going through the checklist INCLUDING making changes in your manuscript *

Approximately 2 additional days

As a result of using this checklist, do you think your manuscript has improved? *

- (selected) yes
- no
- Other:

Would you like to become involved in the CONSORT EHEALTH group?

This would involve for example becoming involved in participating in a workshop and writing an "Explanation and Elaboration" document

- yes
- no
- Other:

Any other comments or questions on CONSORT EHEALTH

Your answer

STOP - Save this form as PDF before you click submit

To generate a record that you filled in this form, we recommend to generate a PDF of this page (on a Mac, simply select "print" and then select "print as PDF") before you submit it.

When you submit your (revised) paper to JMIR, please upload the PDF as supplementary file.

Don't worry if some text in the textboxes is cut off, as we still have the complete information in our database. Thank you!

Final step: Click submit !

Click submit so we have your answers in our database!

Submit

Clear form

Never submit passwords through Google Forms.

This content is neither created nor endorsed by Google. Report Abuse - Terms of Service - Privacy Policy

Google Forms
